# New approach to investigate Common Variable Immunodeficiency patients using spectrochemical analysis of blood

**DOI:** 10.1038/s41598-019-43196-5

**Published:** 2019-05-10

**Authors:** Emma L. Callery, Camilo L. M. Morais, Maria Paraskevaidi, Vladimir Brusic, Pavaladurai Vijayadurai, Ariharan Anantharachagan, Francis L. Martin, Anthony W. Rowbottom

**Affiliations:** 1 Department of Immunology, Lancashire Teaching Hospitals NHS Foundation Trust, Preston, PR2 9HT UK; 2 School of Pharmacy and Biomedical Sciences, University of Central Lancashire, Preston, PR1 2HE UK; 3 University of Nottingham Ningbo China, Ningbo, 315100 China; 4 School of Medicine, University of Central Lancashire, Preston, PR1 2HE UK; 5 Department of Surgery and Cancer, Imperial College London, London, W12 0HS UK; 6 NIHR Lancashire Clinical Research Facility, Lancashire Teaching Hospitals NHS Foundation Trust, Preston, PR2 9HT UK

**Keywords:** Diagnostic markers, Primary immunodeficiency disorders

## Abstract

Common variable immune deficiency (CVID) is a primary immunodeficiency disease, characterized by hypogammaglobulinemia, recurrent infections and various complications. The clinical heterogeneity of CVID has hindered identification of an underlying immune defect; diagnosis relies on clinical judgement, alongside evidence-based criteria. The lack of pathognomonic clinical or laboratory features leads to average diagnostic delays of 5 years or more from the onset. Vibrational spectroscopic techniques such as Fourier-transform infrared (FTIR) spectroscopy have recently gained increasing clinical importance, being rapid-, non-invasive and inexpensive methods to obtain information on the content of biological samples. This has led us to apply FTIR spectroscopy to the investigation of blood samples from a cohort of CVID patients; revealing spectral features capable of stratifying CVID patients from healthy controls with sensitivities and specificities of 97% and 93%, respectively for serum, and 94% and 95%, respectively for plasma. Furthermore we identified several discriminating spectral biomarkers; wavenumbers in regions indicative of nucleic acids (984 cm^−1^, 1053 cm^−1^, 1084 cm^−1^, 1115 cm^−1^, 1528 cm^−1^, 1639 cm^−1^), and a collagen-associated biomarker (1528 cm^−1^), which may represent future candidate biomarkers and provide new knowledge on the aetiology of CVID. This proof-of-concept study provides a basis for developing a novel diagnostic tool for CVID.

## Introduction

Common variable immunodeficiency (CVID) is the most frequent life-threatening and symptomatic primary immune deficiency (PID)^1^. The estimated prevalence is between 1:10,000 and 1:100,000 of the population, with two peak ages of onset, one before the age of ten and another between 30–40 years of age^2,3^. The majority (>80%) of CVID cases are sporadic and the main diagnostic method is exclusion; often with a delay of approximately 5 years^4^. Failure to produce sufficient immunoglobulins results in recurrent infections in 90% of CVID patients; an increased risk of autoimmune disorders (22% of patients) and malignancy (16% of patients)^5–8^. CVID is a heterogeneous group of polygenic disorders for which the exact pathogenesis remains poorly understood^9–11^. Genetic mutations are implicated in CVID in 10–20% of patients; with defects found in more than 30 genes^4,8,12^.

There are no clinical or laboratory features that are pathognomonic for CVID. Diagnostic criteria have therefore been developed which require sequential application of both clinical and laboratory findings in order to increase the specificity of the diagnosis. Current diagnostic criteria^5,13,14^ define hypogammaglobulinemia as a major requirement for the diagnosis of CVID, but only when used in conjunction with further clinical or laboratory findings. This is due to the low diagnostic specificity of hypogammaglobulinemia for CVID; as reduced serological levels of immunoglobulin are also associated with a vast array of other primary and secondary immune disorders. The incidence and prevalence rates of hypogammaglobulinemia are not clearly defined, however secondary causes are more common^15^. Although the requirement to exclude secondary causes of hypogammaglobulinemia limit the use of this finding as a diagnostic marker for CVID, it remains a fundamental test, given that the hallmark of the disease is a reduced serum level of IgG, IgA and/or IgM. The definition of local or regional population reference ranges will impact the diagnostic utility of immunoglobulin results, as will the laboratory choice of analytical method (nephelometric and tubidmetric methods are most widely used). An absolute lower limit value of IgG at 4.5 g/L for adults has been proposed, as despite the wide range of IgG levels observed in CVID patients, Chapel and Cunningham-Rundles^2^ described the majority of their 334 patients (94.2%) as having initial IgG levels <4.5 g/l at diagnosis.

Further laboratory testing, such as measuring specific antibodies to vaccine responses, enumeration of lymphocyte subsets (B, T and NK cells) and class-switched memory B cells by flow cytometry, can provide additional evidence to suggest defective antibody production. Findings are variable across the disease group and also within individual patients. Repeat testing has therefore been suggested to confirm any sub-normal findings; these limitations have been discussed in recent diagnostic criteria^5,13^.

Efforts to categorise clinical subgroups within cohorts of established CVID patients using flow cytometry have further demonstrated the complex aetiology of this disorder, emphasising the variety of B cell differentiation defects that can contribute to the disease. Phenotypic analysis of B cells using population sizes of class-switched memory- and transitional B cells, in correlation with clinical aspects has generated three classification protocols for patients with CVID^16–18^. Whilst these studies demonstrate that B cell homeostasis is a pathogenic and clinically meaningful parameter for classification, reduced numbers of class-switched or memory B cells are not specific to CVID hence the diagnostic utility of these tests in isolation is limited^2^.

Further challenges with current diagnostics relate to a lack of understanding as to which physiological compartment should be investigated for CVID-associated abnormalities, *i.e*., tissues, biological fluids, or cells. A potential novel diagnostic methodology for CVID is vibrational biospectroscopy. High resolution spectroscopy methods such as Fourier-transform infrared (FTIR) spectroscopy can provide unique spectral patterns that reflect the chemical and molecular composition of biological samples. We hypothesised that pathological changes in CVID patients produce characteristic FTIR spectra that distinguish them from healthy controls. The interaction of infrared (IR) light with biological matter produces an absorption plot, or ‘spectral fingerprint’, for each biological sample. The principles and biological applications of FTIR spectroscopy have been reviewed in detail in Baker *et al*.^19^. Vibrational spectroscopy is gaining recognition in the field of diagnostic medicine for a range of complex pathologies, mostly for malignancies^19–21^. A key requirement for diagnostic investigations is the highly accurate discrimination of pathological features from healthy neighbouring tissue or cells. These measurements are often performed on samples characterised by high background signals (or ‘noise’) relating to biological activity (*e.g*., increased cell turnover or inflammatory states). Due to the high complexity of vibrational spectroscopy data, computational-based methods (chemometrics) are needed to explore and extract relevant information from the experimentally acquired spectra. For this, multivariate classification techniques can be employed for feature extraction and classification, allowing biochemically-relevant information to be extracted and the automatic grouping of samples into pre-defined categories. This can be achieved using a combination of chemometric algorithms, such as forward feature selection (FFS), principal component analysis linear discriminant analysis (PCA-LDA), and principal component analysis support vector machines (PCA-SVM). All of these algorithms are based on a principal component analysis (PCA) decomposition, which significantly reduces the original data complexity to a fewer number of relevant factors, named principal components (PCs). PCA-LDA performs a linear discriminant analysis (LDA) of the PCA scores to assign the samples to their predicted groups; whereas PCA-SVM does the same procedure but in a non-linear classification fashion through a support vector machines (SVM) algorithm. FFS allows identification of main biomarkers responsible for class differentiation by calculating p-values for the spectral wavenumbers with larger PCA loadings. Vibrational spectroscopy has been successfully applied across a wide area of clinical medicine; providing a new approach to detect molecular and structural changes caused by complex disorders such as Alzheimer’s disease^22,23^, multiple sclerosis^24^, mental disorders^25,26^, HIV/AIDS^27^, diabetes^28^ and carcinogenesis^25,29–32^. High diagnostic accuracy was demonstrated for classification of numerous cancer types and other biological applications^33–38^. We hypothesised that vibrational spectroscopy will demonstrate similar analytical advantages within our cohort, allowing for the sensitive detection of characteristic spectral fingerprints that represent underlying pathological processes in CVID patients.

To our knowledge, this study reports for the first time, the application of FTIR methods, specifically, attenuated total reflection-FTIR (ATR-FTIR) spectroscopy, for detection of CVID. In this first phase we have explored the application of this technique to stratify CVID patients from healthy controls (HC) using serum and plasma. We performed stratified spectroscopic classification at multiple levels, to differentiate subgroups of CVID patients with- and without- further clinical complications. Finally, we have identified a number of meaningful and discriminating spectral biomarkers, tentatively assigned to specific molecular entities. These promising initial findings encourage further development of FTIR spectroscopy as a diagnostic technique for immune deficiency.

## Results

The major aim of this study was the discrimination of CVID patients from HC in two biofluids; serum and plasma using FTIR-spectroscopy and multivariate analysis techniques. The ATR-FTIR spectra from 51 subjects (1020 spectra) were obtained and analysed using multiple chemometric methods. An exploratory (unsupervised) analysis using PCA model was undertaken, followed by classification using supervised methods (PCA-LDA, FFS, SVM) to enable successful segregation of subjects into their respective groups, CVID patients and healthy controls.

## Discrimination of CVID patients from Healthy controls

### Analysis of prominent IR spectral regions reveals significant variance between CVID and HC groups

Rubber-band baseline correction and vector normalisation produces spectra for crude visualisation of differences between the two groups, and corrects for experimental variation; this recognised technique improves accuracy and interpretability of the data whilst maintaining spectral integrity. The generated figures (Supplemental Fig. 1a,b (fingerprint region); Supplemental Fig. 1e,f (high region)) demonstrate visual spectral similarities for each class (CVID, HC), prior to the application of multivariate analysis tools. As expected for biological samples, the Amide I band was most prominent in the IR spectrum of the fingerprint region, dominated by C=O stretching, and N-H bending vibrations of proteins^39,40^. To enhance spectral variability between groups, second-order differentiation was applied (Supplemental Fig. 1c,d (Fingerprint region); Supplemental Fig. 1g,h (High region)) prior to implementation of multivariate approaches.

#### Serum

Key differences observed in the fingerprint region of serum (Supplemental Fig. 1a) at the pre-processing stage were lower absorbance intensities in the CVID group compared to HC at the nucleic acid-associated asymmetric stretching (ν_as_) of PO_2_^−^ (DNA/RNA) [1242 cm^−1^ (p = 0.0003). At the high region (Supplemental Fig. 1e) the CVID group revealed increased absorbance peaks within the lipid and protein associated (CH_3_ and CH_2_) stretching vibrations^21^ (serum 3000–2800 cm^−1^ p =< 0.0001).

#### Plasma

The fingerprint region of the plasma spectra (Supplemental Fig. 1b) revealed greatest variance between the two groups at the Amide I [1643 cm^−1^ (p < 10^−6^)] and Amide II [1535 cm^−1^ (p < 10^−6^)] and Amide III peaks [1315 cm^−1^ plasma p =< 0.0001)], with lower absorbance found in the CVID group compared to HC. At the high region (Supplemental Fig. 1f), peak increases at 2928–2932 cm^−1^ (p =< 0.0001) were found in the CVID group compared to HC.

### Segregation of CVID patients from HC can be demonstrated following multivariate analysis of the entire spectral dataset for both Fingerprint (1800–900 cm^−1^) and High (3700–2800 cm^−1^) regions

**Figure 1:**
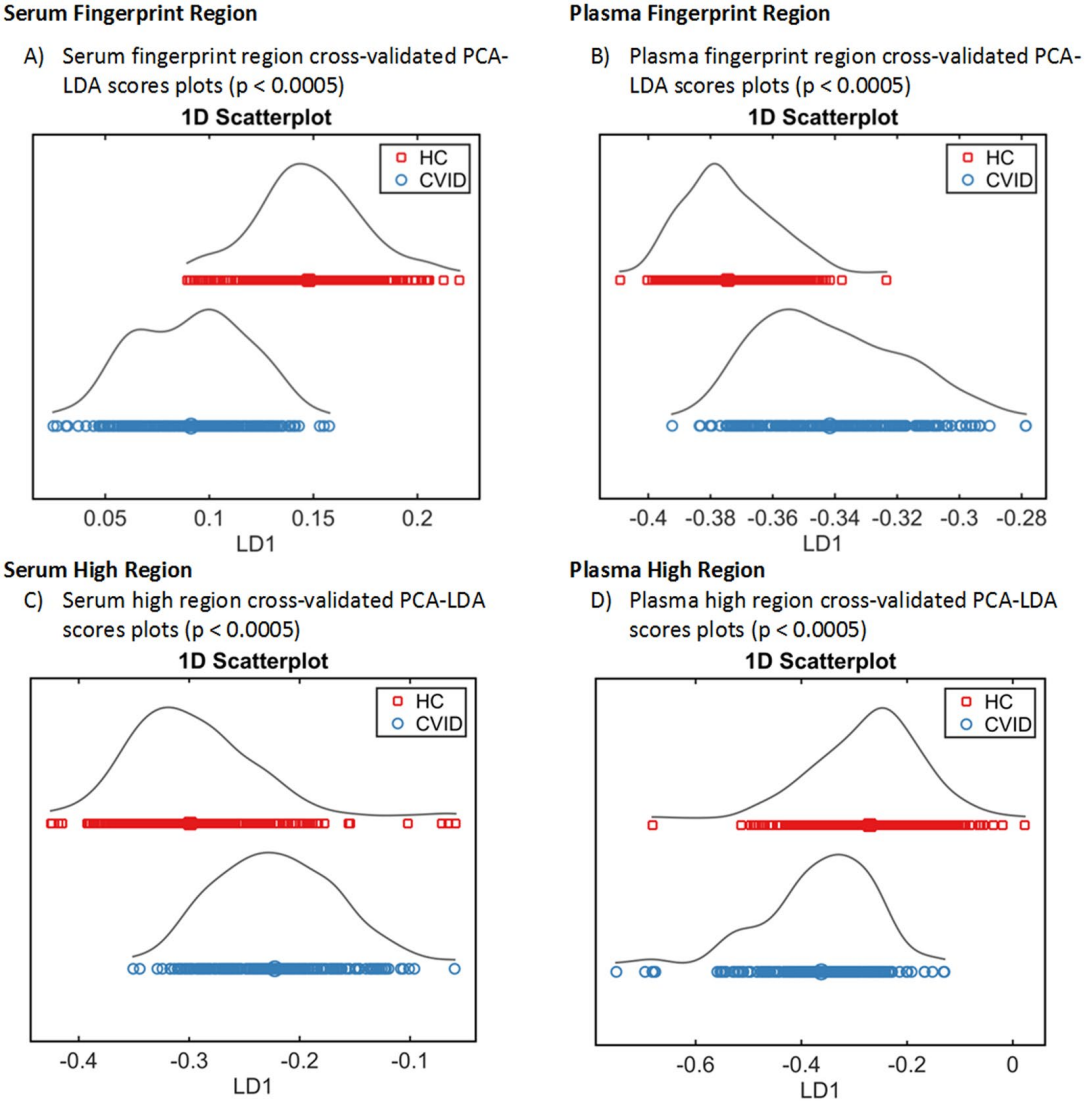
Supervised multivariate analysis techniques (PCA-LDA) successfully segregate classes (CVID vs HC). (**A**,**B**) Fingerprint region (900–1800 cm^−1^); 1D scores plots (LD1) after cross-validated PCA-LDA of the training dataset (CVID *n* = 13; HC *n* = 18) for serum and plasma respectively. (**C**,**D**) High region (2800–3700 cm^−1^); 1D scores plots (LD1) after cross-validated PCA-LDA of the training dataset (CVID *n* = 13; HC *n* = 18) for serum and plasma respectively.

Additional examination of the spectra was performed using cross-validated PCA-LDA. The 1D PCA-LDA scores plots (Fig. 1A–D) were generated, and utilised to illustrate the significant differences between the CVID group (red) and the HCs (blue) (p < 0.0005); the “scores” here represent individual spectra, (fingerprint region serum p < 10^−6^ and plasma p < 10^−6^; high region serum p < 10^−6^ and plasma p ≈ 10^−4^). To further explore whether the classes could be significantly separated on a study subject-level basis, mean values of the 20 (second-order differentiated) spectral replicates per sample were calculated prior to performing cross-validated PCA-LDA (illustrated in Supplemental Fig. 1a–d), in which each score represents an individual study subject. We again demonstrated significant differences between HC and CVID groups, this time on a patient level, for both serum and plasma at the fingerprint region (p < 10^−6^ for both), and at the high region (p < 10^−6^ for both).

## Discrimination of CVID patients from HC using classification models

### SVM classification prior to incorporation of additional clinical information

**Figure 2:**
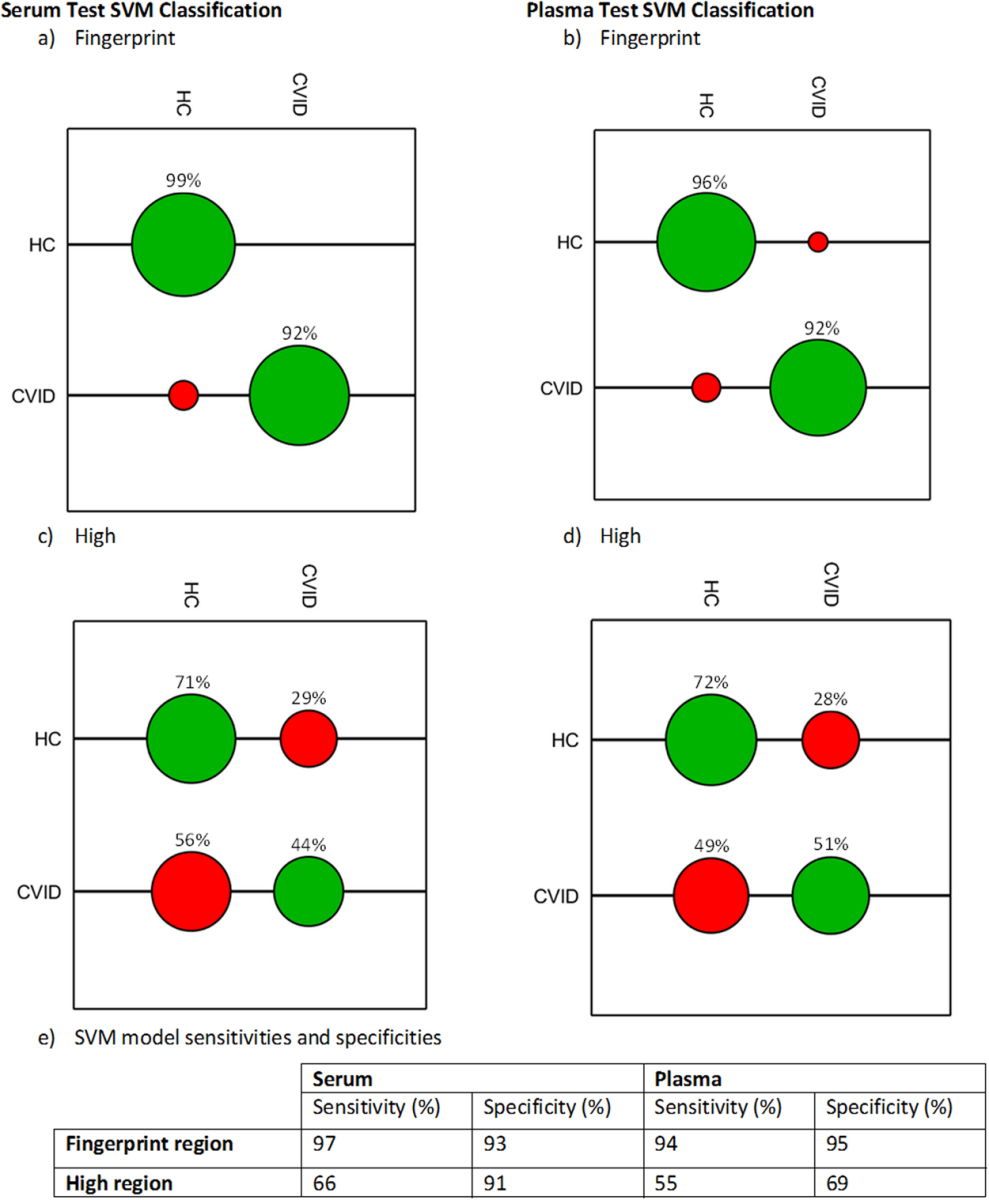
SVM classification model for CVID vs HC using each biofluid at the fingerprint (900–1800 cm^−1^) and high region (2800–3700 cm^−1^) of the spectrum. (**a**–**d**) SVM confusion matrices for (**a**) serum fingerprint, (**b**) plasma fingerprint, (**c**) serum high and (**d**) plasma high regions. The tuning parameters (c, γ) extracted from a grid search of the training dataset were used to subsequently generate confusion matrices (coloured balls) and associated classification rates for the test dataset (CVID *n* = 8; HC *n* = 12). (**e**) Sensitivity and specificity of SVM models calculated using the corresponding ‘accumulated hits’ data (individual spectra).

Following successful segregation of classes using PCA-LDA scores plots, the ability and performance of FTIR as a tool to discriminate CVID patients from HC was assessed through creation of classification models. Classification of CVID and HC was performed on the Fingerprint and High regions of the IR spectrum using support vector machine (SVM) learning algorithms as described in the methods. The SVM models were generated using 2/3 of the spectral data for the four distanced groups (Serum Fingerprint, Serum High, Plasma Fingerprint and Plasma High) prior to being tested with the remaining 1/3. The confusion matrices generated following the input of the test data into each classification model can be illustrated graphically in the form of confusion balls (Fig. 2a–d). Supplemental Table 2 specifies the *c* and γ values outputted by the four grid searches. Within the serum, correct classification was achieved for 99% of HC and 92% of CVID patients using the fingerprint region (Fig. 2a); and 71% of HC and 44% of CVID patients using the high region (Fig. 2c). Within the plasma, correct classification was achieved for 96% of HC and 92% of CVID patients using the fingerprint region (Fig. 2b); and 72% of HC and 51% of CVID patients using the high region (Fig. 2d). The highest sensitivities and specificities were obtained for the fingerprint region, achieving 97% and 93% respectively for serum; 94% and 95% respectively for plasma. In the high region, sensitivities and specificities were lower, at 66% and 91% respectively for serum; 55% and 69% for plasma (Fig. 2d).

### SVM classification subsequent to incorporation of clinical subgrouping

**Figure 3:**
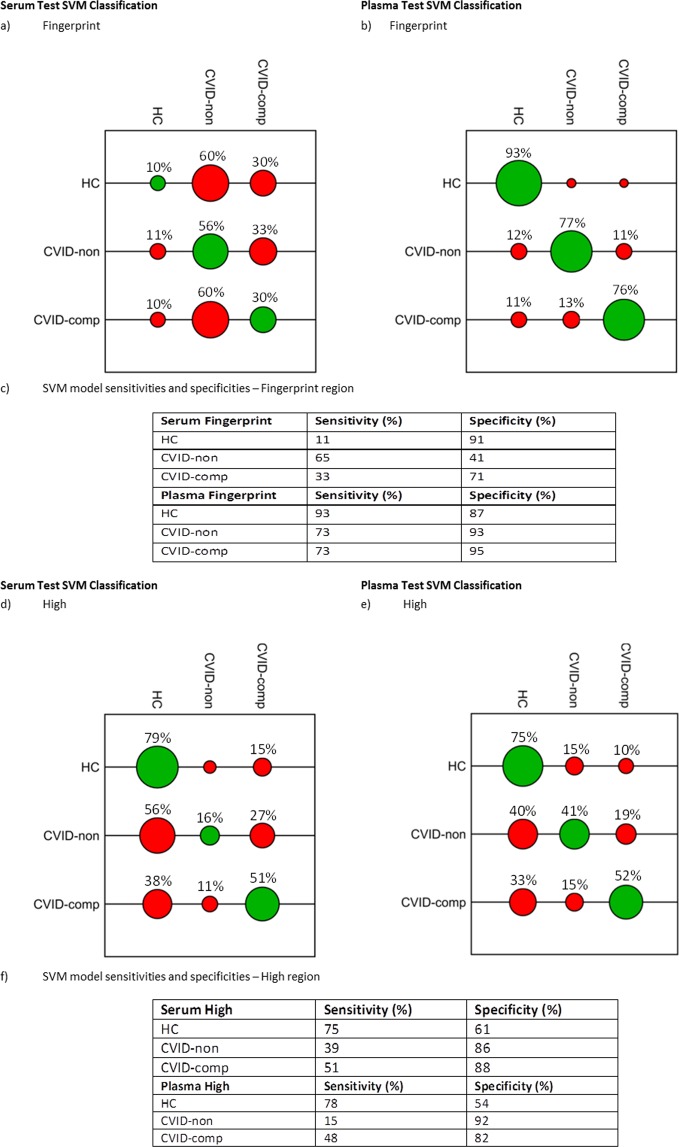
SVM classification model ‘HC vs CVID-non complications vs CVID-complications’ for each biofluid at the fingerprint (900–1800 cm^−1^) and high region (2800–3700 cm^−1^) of the spectrum. (**a**,**b**) SVM confusion matrices for a, serum fingerprint, (**b**) plasma fingerprint. The tuning parameters (**c**, γ) extracted from a grid search of the training dataset were used to subsequently generate confusion matrices (coloured balls) and associated classification rates for the test dataset (CVID *n* = 8; HC *n* = 12). (**c**) Sensitivity and specificity of SVM models using Fingerprint region, calculated using the corresponding ‘accumulated hits’ data (individual spectra). (**d**,**e**) SVM confusion matrices for (**c**) serum high and (**d**) plasma high regions. (**e**) Sensitivity and specificity of SVM models using High region, calculated using the corresponding ‘accumulated hits’ data (individual spectra).

CVID patients were further divided based on clinical manifestations (see methods) prior to application of SVM learning algorithms for classification (Fig. 3); the parameters identified during grid searches of the training data are included in Supplemental Table 3.

#### Serum

In the HC group, 10% of subjects were classified correctly; within the two CVID sub-groups, the classification model correctly assigned 56% of the CVID patients without further complications and 56% of patients with further complications into their respective groups (Fig. 3a). Classification using the high region achieved correct group assignment for 79% of HC subjects, 16% of CVID patients without further complications and 51% of CVID patients with further complication (Fig. 3d). The sensitivities and specificities of the fingerprint SVM model after incorporation of clinical data are documented in Fig. 3c; with greatest specificity, 91%, achieved when classifying HC subjects using the fingerprint region. For the classification of CVID subgroups using the high region, specificities of 92% for CVID-non complication patients and 82% for CVID-complication patients were achieved (Fig. 3f). In comparison to the classification model based on HC vs all CVID patients, sensitivities achieved following clinical subgrouping were lower, at 41% for CVID-non complication and 71% for CVID-complication patients using the fingerprint region.

#### Plasma

SVM models generated for the plasma data demonstrated increased classification ability compared to the serum, correctly assigning 93% of HC, 77% of CVID-non complication patients and 76% of CVID-complication patients using the fingerprint region (Fig. 3b); classification rates in the high region were 75%, 41% and 52% respectively (Fig. 3e). Sensitivities and specificities for the three groups are documented in (Fig. 3c,f). Classification using the fingerprint region achieved highest sensitivities and specificities; HCs were classified with a sensitivity and specificity of 93% and 87% respectively, CVID-non complication patients with 73% and 93% respectively and CVID-complication patients with 73% and 95% respectively.

## Biomarker analysis

**Figure 4:**
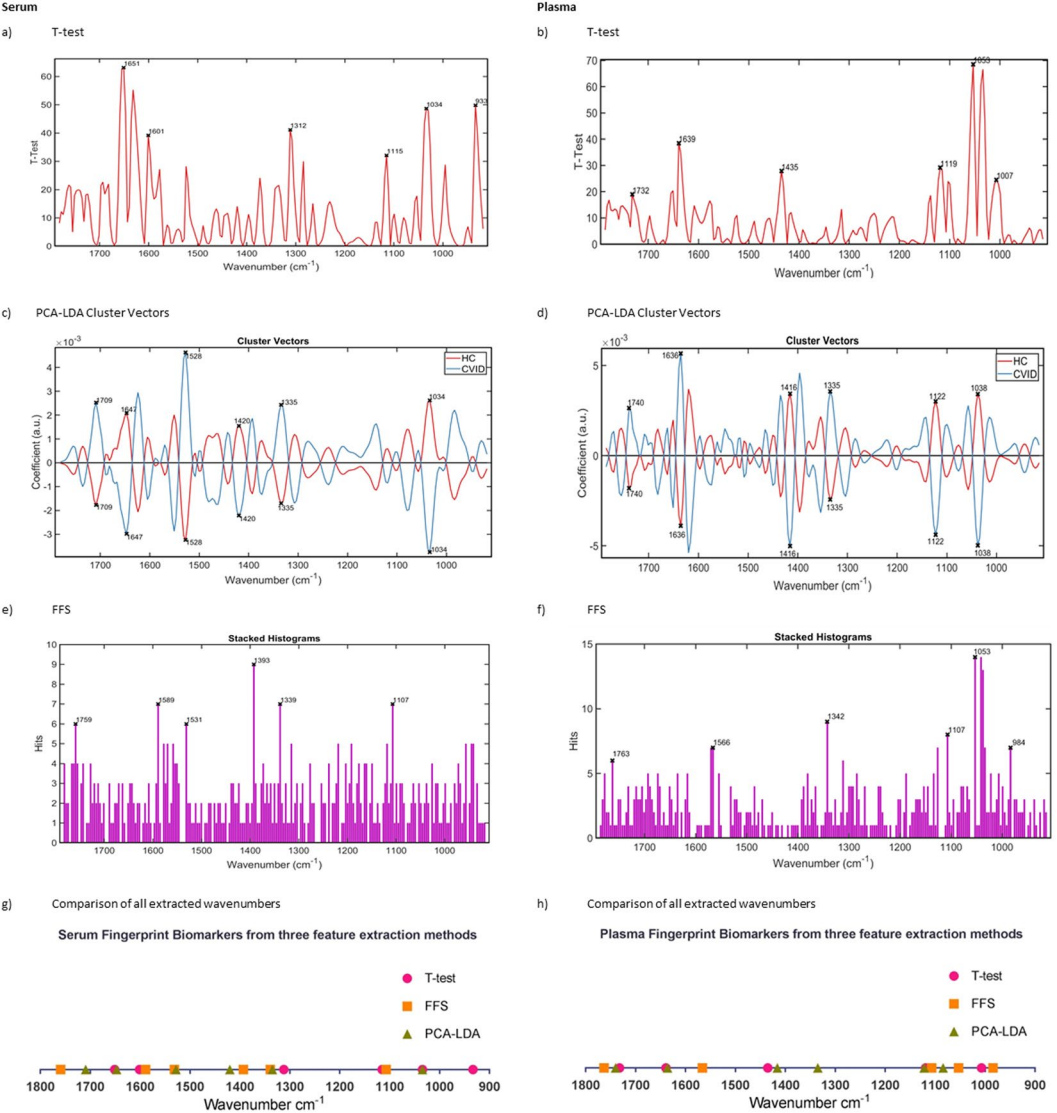
Serum and plasma Fingerprint region biomarkers identified using three feature extraction methods on the training dataset (CVID *n* = 13; HC *n* = 18). (**a**) Serum T-test. (**b**) Plasma T-test. (**c**) Serum FFS. (**d**) Plasma FFS. (**e**) Serum cross-validated PCA-LDA. (**f**) Plasma cross-validated PCA-LDA. (**i**,**j**) Visual representation of wavenumber location for extracted biomarkers from each method for serum and plasma respectively. FFS Forward Feature Selection.

Feature extraction was performed as described in the methods; the key biomarkers extracted from each technique are illustrated in Fig. 4 (fingerprint) and Supplemental Fig. 3 (high region) and documented in Supplemental Tables 4 and 5, along with the tentative molecular assignments previously described for individual wavenumbers. Relative increases or decreases in the absorbance intensity of CVID spectra are indicated where further subject-level analysis was performed.

Figure 4 and Supplemental Fig. 3(a,b) illustrates the 6 most variant peaks elucidated using the Student’s T-Test method; Fig. 4 and Supplemental Fig. 3(c,d) shows the biomarkers selected from the cluster vectors using PCA-LDA; and Fig. 4 and Supplemental Fig. 3(e,f) are FFS histograms illustrating the number of times each wavenumber was selected as a key feature for differentiating between the two classes. Figure 4 and Supplemental Fig. 3(g,h) visually represent the spectral wavenumber location where the 18 biomarkers were found using the three techniques. The points showing close proximity or overlap indicate the close agreement of the selected wavenumbers from the three methods of biomarker extraction.

### Fingerprint region biomarkers

#### Serum

Within the serum a total of 10 spectral wavenumbers (p =< 0.05) were extracted. Wavenumber 1034 cm^−1^ was extracted by two different methods, which strengthens its utility as a serum biomarker.

**Figure 5:**
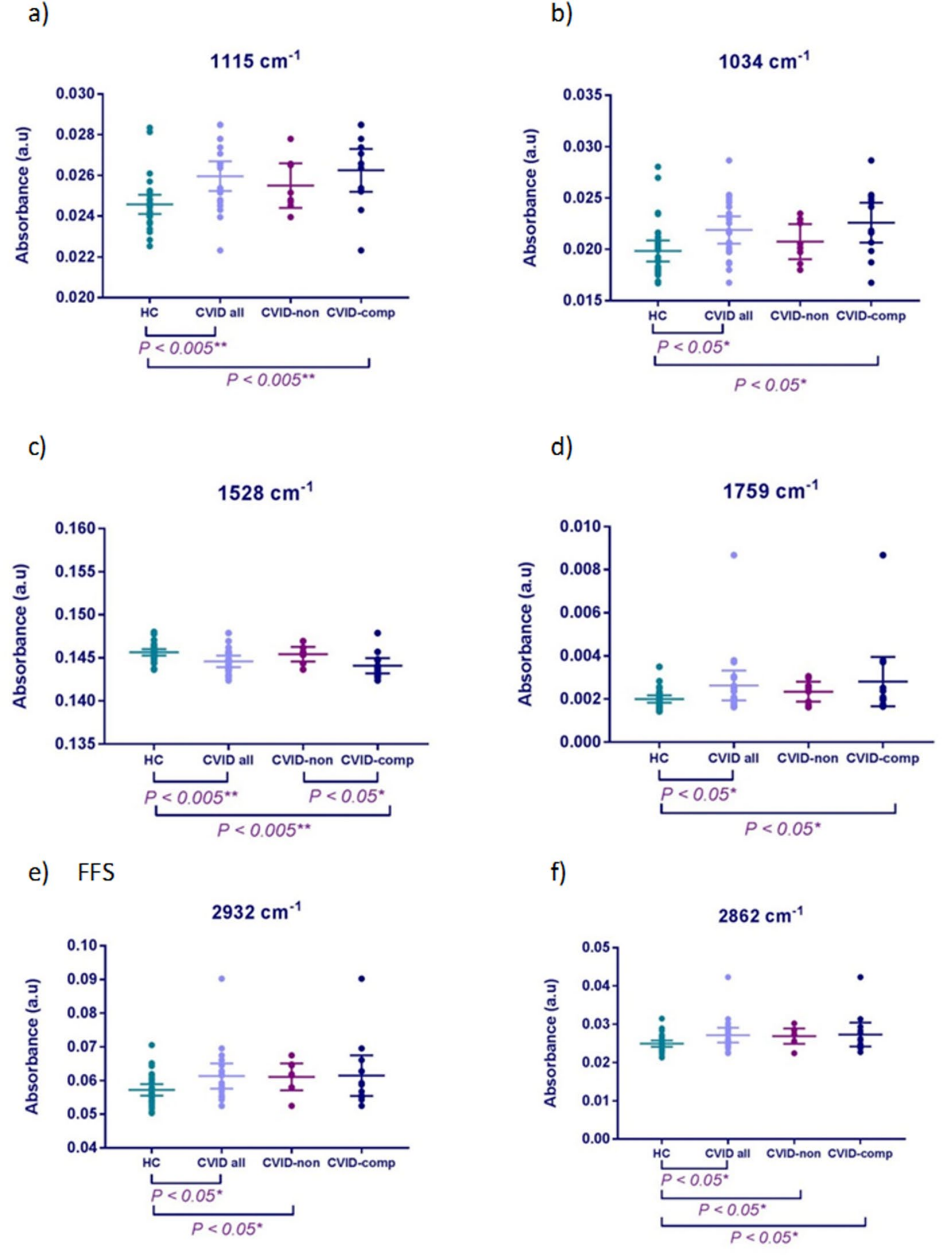
Serum Biomarkers. Between group absorbance intensity analysis for discriminating spectral wavenumbers. Mean absorbance intensity plotted for each study subject (20 replicates) per wavenumber. CVID *n* = 21 (CVID-non *n* = 8; CVID-comp *n* = 13); HC n = 30. Data are expressed as mean (±95% CI). *P < 0.05; **P < 0.005.

Individual subject-level wavenumber intensity analysis was performed on each of the biomarkers (pre-processed (rubber-band, vector normalised) data). Four key peaks were shown to demonstrate significant differences (p < 0.05) between mean absorbance intensity for CVID and HC subjects (Fig. 5a–d); 1115 cm^−1^ (symmetric stretching P–O–C), 1034 cm^−1^ (collagen), 1528 cm^−1^ (C=N guanine, adenine, cytosine) and 1759 cm^−1^ (C=O ester group vibration of triglycerides). Intensity differences between the clinical sub-groups (patients with- and without further complications), were also explored. The increased absorbance intensity of wavenumbers 1115 cm^−1^, 1034 cm^−1^ and 1759 cm^−1^ were statistically significant in CVID compared to the HC group; whereas the intensity of wavenumber 1528 cm^−1^ was lower. This finding was mirrored in the CVID subgroups; patients with further complications demonstrated higher absorbance intensity for 1115 cm^−1^, 1034 cm^−1^ and 1759 cm^−1^ compared to the patients without complications; with lower intensities observed for wavenumber 1528 cm^−1^.

#### Plasma

**Figure 6:**
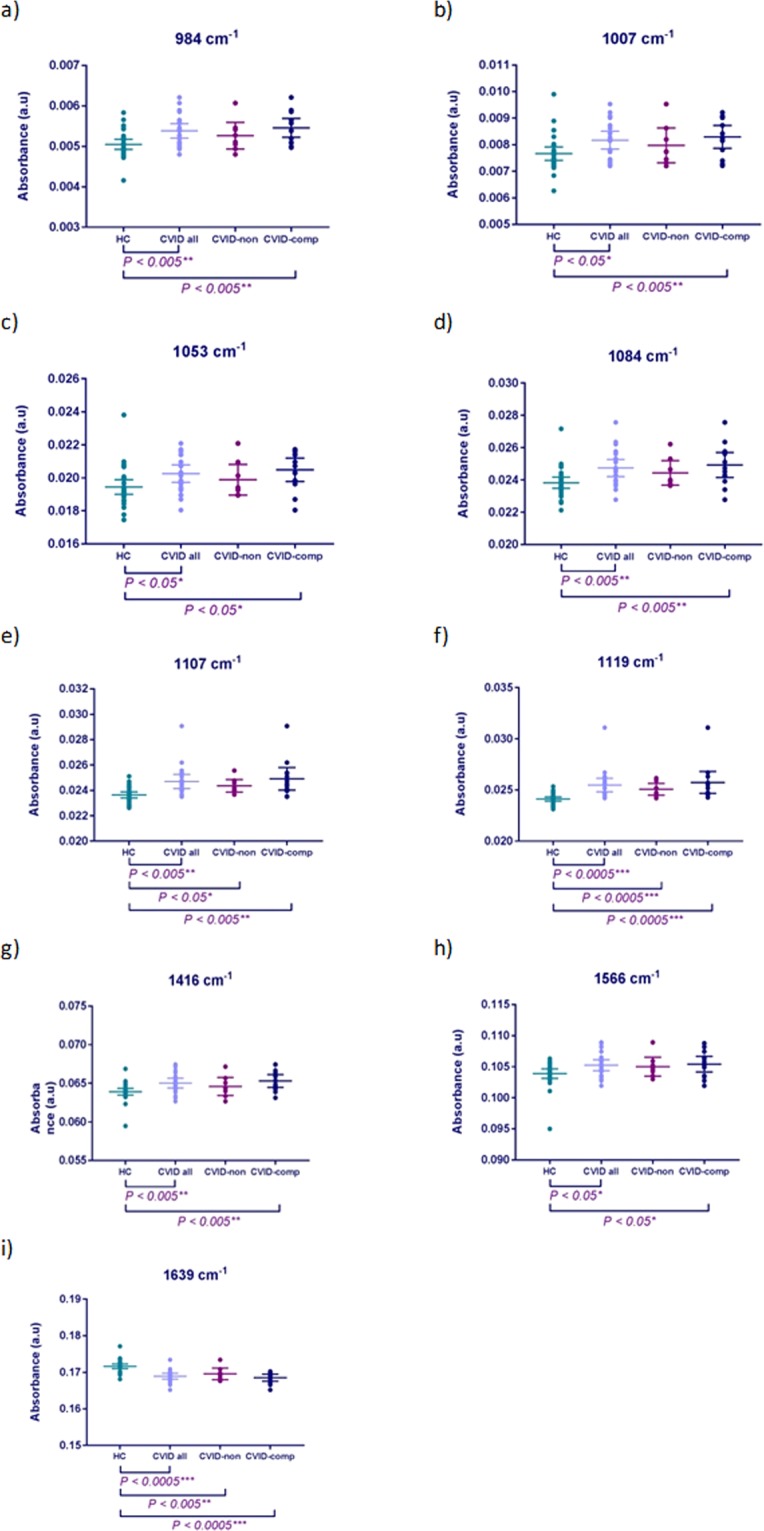
Plasma Biomarkers. Between group absorbance intensity analysis for discriminating spectral wavenumbers. Mean absorbance intensity plotted for each study subject (20 replicates) per wavenumber. CVID *n* = 21 (CVID-non *n* = 8; CVID-comp *n* = 13); HC *n* = 30. Data are expressed as mean (±95% CI). *P < 0.05; **P < 0.005; ***P < 0.0005.

12 unique wavenumbers demonstrated significant absorbance intensity differences (p =< 0.05) within the plasma when comparing CVID patients and HC (Supplemental Table 4). Of these, 9 wavenumbers revealed significant differences on individual subject-level analysis (Fig. 6a–i); 984 cm^−1^, 1007 cm^−1^, 1053 cm^−1^, 1084 cm^−1^, 1107 cm^−1^ and 1119 cm^−1^ (within phosphodiester region 900^−1^ 300 cm^−1^); 1416 cm^−1^, 1566 cm^−1^ and 1639 cm^−1^ (within protein region; Amide I, Amide II). Wavenumber 1053 cm^−1^ was extracted by two different methods, strengthening its utility as a serum biomarker.

Absorbance intensities of 8 of the 9 wavenumbers increased in the CVID group compared to HC, with only wavenumber 1639 cm^−1^ (Amide I; thymine, adenine, guanine) demonstrating a lower absorbance intensity. Consistent with the serum data, the findings within the CVID subgroups reflected the intensity differences observed between the CVID patients and HC, with absorbance intensity for 8 of the 9 biomarkers found to be increased in the CVID patients with further complications compared to those patients without, and only wavenumber 1639 cm^−1^ demonstrating a lower intensity respectively.

Differences between HC subjects and CVID patients with further complications were calculated as significant for all 9 biomarkers. In contrast to the serum data, exploration of the plasma biomarkers revealed significant differences between HC and CVID patients without further complication. Wavenumbers 1107 cm^−1^, 1119 cm^−1^ and 1416 cm^−1^ all demonstrated higher absorbance intensities in the CVID-non group compared to HC, whereas wavenumber 1639 cm^−1^ showed lower intensity. None of the 9 plasma wavenumbers demonstrated significant absorbance differences to segregate the two CVID subgroups.

### High region biomarkers

#### Serum

In the high region 11 biomarkers were calculated to be statistically significant when comparing intensities between the two classes (HC vs CVID) (Supplemental Table 5).

On individual subject-level analysis, 2 significant biomarkers (p < 0.05) were identified; 2932 cm^−1^ and 2862 cm^−1^, corresponding to important CH, CH_2_ and CH_3_ molecular vibrations found in lipids and fatty acids (Fig. 5e,f). The absorbance intensity of both wavenumbers were increased within the CVID patients compared to HC; similarly, intensity increases were observed in CVID patients with further complications compared to those without. The increased absorbance intensities were calculated to be significant for HC vs CVID patients-with further complications, HC vs CVID patients-without complications, but not between the two CVID subgroups.

#### Plasma

10 biomarkers were found to demonstrate significant differences between CVID and HC groups (Supplemental Table 5). Only one wavenumber demonstrated significant intensity differences between HC and CVID patients on an individual subject level (3302 cm^−1^) however the tentative assignment of this wavenumber to water renders it unsuitable for use as a biomarker.

### Discriminating biomarkers between the CVID subgroups

Of all identified serum and plasma biomarkers (total 17) only one wavenumber, 1528 cm^−1^ (Fig. 5c) detected in the fingerprint region of the serum, was shown to be a discriminating biomarker between the CVID patients presenting with further complications (CVID-comp) and those remaining complication-free (CVID-non) (p = 0.037). The mean intensity of this wavenumber was reduced in both of the CVID groups compared to HC (HC = 0.1456, CVID-non = 0.1454, CVID-complications = 0.1455).

## Discussion

Although great progress has been made in developing diagnostic and classification criteria for CVID^6,16–18,41^, there is still no robust method to achieve this. This study has demonstrated the effectiveness of FTIR spectroscopic methods towards the diagnosis of CVID, correctly segregating CVID patients and HC into their respective groups following analysis of biofluids. This has been performed using a classification model on the fingerprint region of the ATR-FTIR spectrum with a sensitivity and specificity of 94% and 95% respectively for plasma, and 97% and 93% respectively for serum. The high region of the spectrum was similarly analysed, providing a classification model with a sensitivity and specificity of 55% and 69% respectively for plasma, and 66% and 91% respectively for serum; suggesting analysis of the fingerprint region would be more appropriate for classification of CVID. The use of blood-based vibrational spectroscopy to detect differences between clinical sub-groups of CVID patients has also been achieved; demonstrated by the successful assignment of individual study subjects into their respective groups; greatest sensitivities and specificities were achieved within the plasma fingerprint region, at 93% and 87% respectively for HC subjects, 73% and 94% for CVID patients without complications and 73% and 95% for CVID patients with further complications.

A further aim of the current study was to extract spectral biomarkers responsible for the differentiation between CVID patients and HC. As each wavenumber corresponds to molecular bonds within biochemical sample components, we could tentatively assign the most discriminating peaks for use as potential disease biomarkers. In the serum we found evidence of detectable alterations in band intensities at four wavenumbers 1034 cm^−1^ (collagen^42^), 1115 cm^−1^ (symmetric stretching P–O–C^43^), 1528 cm^−1^ (C=N guanine, adenine, cytosine^43,44^) and 1759 cm^−1^ (C=O ester group vibration of triglycerides^45,46^). We hypothesise that the significant increase in the collagen-associated peak (1034 cm^−1^) observed in CVID patients may be associated with increased collagen turnover and production of degradation fragments following recurrent respiratory tract infection-driven lung damage. Abnormal distributions of extracellular matrix components, such as collagen type I and III, have been demonstrated in fibrotic lung conditions such as alveolitis, respiratory distress syndrome and chronic obstructive pulmonary disease (COPD)^47–50^; with degradation fragments detected and used as diagnostic makers in COPD and idiopathic pulmonary fibrosis^51^. We postulate 1034 cm^−1^ wavenumber analysis could have similar clinical utility, thus warranting a more detailed evaluation as a disease marker. Interestingly, in patients with further CVID-associated complications, a further increase in 1034 cm^−1^ peak intensity was observed, significantly segregating this group from the HCs (p < 0.05). This increase may signal the progression of early lung damage to bronchiectasis; if so, this marker could be used to identify high risk patients and avoid progression of this irreversible complication reported in over 2/3 of CVID patients^3^. Given the ubiquitous expression of collagen throughout the body, biomarker 1034 cm^−1^ intensity could reflect systemic serological levels, thus, further exploration in patients with other fibrotic co-morbidities or respiratory disorders are required to determine disease specificity.

Examination of nucleic acid-associated wavenumbers revealed a number of interesting observations. Three significant wavenumbers observed in plasma showed an increased intensity in CVID patients compared to HCs (984 cm^−1^, 1053 cm^−1^, 1084 cm^−1^) as did wavenumber 1115 cm^−1^ in serum. Each of these wavenumbers have been assigned to bond vibrations found in molecules containing phosphodiester regions, PO_2_ and P-O-C bonds, such as moieties found in the DNA/RNA sugar phosphate backbone^42,44,45,52–58^. Wavenumber intensity increases were accentuated further in the CVID-complications group compared to those patients without complications; albeit not to a statistically significant level (p => 0.05). We postulate that the increased trend in DNA/RNA-associated wavenumbers observed in the CVID group may be associated with increased levels of transcription for a number of genes involved in immune signalling pathways. Altered cytokine and chemokine profiles have been observed in CVID patients by several groups^59–65^, thought to be driven by a state of chronic immune activation. This has been attributed to microbial activation of monocyte-macrophage and granulocyte lineages^59^ or alternatively a predominance of the Th2 pathway^60^. CVID-associated profiles include increased serum levels of IL-4 and IL-10^60^, IL-2 and IL-10^61^, IL-6, IL-8, IL-1RA and TNF-a^62^ and increased IL-10, IL-RA, and TNF-α^63^. Treatment with IVIg has been shown to dampen down this immune activation in CVID patients, although the exact mechanism of action remains unclear^59^. Of note within our study, we found CVID patients with further complications demonstrated a further intensity increase in the four DNA/RNA-associated wavenumbers. One suggestion for this could relate to poor control of immune activation due to higher requirements of replacement Ig in certain individuals. Further investigation into elevated serological levels of chemokines and cytokines in relation to key wavenumber intensity is warranted to determine the relationship between these CVID-associated biomarkers.

Surprisingly, wavenumbers 1528 cm^−1^ (in serum) and 1639 cm^−1^ (in plasma) were shown to have a decreased absorbance intensity in CVID patients compared to HC (p < 0.005 and p < 0.0005, respectively). These wavenumbers have been assigned more closely to C=N, C=C and N-H bonds found in nucleotide bases such as guanine, adenine, cytosine and thymine^37,44,66^, as opposed to phosphate-associated moieties as in the previously described DNA/RNA-associated wavenumbers. We speculate this may reflect a general decrease in the nucleotide pool in CVID patients, and if so may elucidate some of the far-reaching biological and metabolic effects observed within this group. Since DNA replication is pertinent to chromosomal replication, amongst numerous other activities, these may all be directly or indirectly affected when nucleotide concentrations deviate from a physiologically normal range^67^. Unbalanced nucleotide pools have been linked to the activation of p53 and cell cycle arrest in actively dividing cells^68^, therefore similar processes could relate to B cell maturational arrest in CVID.

The intensity in both wavenumbers 1528 cm^−1^ and 1639 cm^−1^ were further decreased in the CVID subgroup with further complications, compared to those without complications. Of heightened interest, wavenumber 1528 cm^−1^ was the only biomarker of the 18 identified to demonstrate significant differences in absorbance intensity between the two CVID subgroups (p < 0.05). This wavenumber could aid further elucidation of disease pathophysiology in addition to serving as a potential marker to determine severity of disease and development of further complications.

The findings from the first stage of this study are encouraging based on the impact that the translation of FTIR spectroscopy into a diagnostic platform for CVID could have on clinical practice. Whilst we have demonstrated the ability of this method to correctly classify CVID patients from HCs, diagnostic capabilities must be further established in subsequent multi-centre studies.

Within the setting of CVID, the diagnostic efficiency of current laboratory methods and FTIR spectroscopy will remain difficult to ascertain until disease-specific features and pathogenic disease mechanisms are further elucidated. The low diagnostic specificity of current tests such as serum immunoglobulins, vaccine responses and B cell immunophenotyping, and reliance on complex classification criteria for CVID, highlights the clinical requirement for an improved approach. In order to demonstrate the power of biospectroscopy as a novel diagnostic tool, the next phase of this study will work towards the validation and verification of the method. Test specificity will be addressed through inclusion of additional patient groups, across multiple centres, such as patients with other primary- or secondary immune deficiency disorders. Further work to fully determine how the identified FTIR biomarkers relate to the molecular and cellular composition of CVID patient samples will be a key milestone in determining whether any pathognomonic features can be identified. Until this is achieved, it is most likely that FTIR will be used alongside current diagnostic methods in order to add a further level of evidence-based criteria to the diagnosis of CVID.

Once achieved, a major advantage of using FTIR over current methods would be the capability for monitoring multiple biochemical changes in patient samples over a time-course analysis. The information collected would enable clinicians to adapt treatment options and undertake additional investigations in a timely manner. By detecting disease-associated complications earlier, before irreversible damage occurs, the life-expectancy of this patient group (in which secondary complications have the biggest impact), could potentialy be extended^8^. In contrast to some of the limitations facing FTIR spectroscopy analysis in other high-risk clinical areas such as malignancy, where it is often required as a one-shot investigation, CVID is a chronic, life-long condition and therefore would be a more suitable candidate for long-term monitoring.

In conclusion, our study has demonstrated that FTIR spectroscopy is a promising analytical tool for determining differences between healthy controls and CVID patients. A classification method based on the fingerprint region in serum was able to correctly discriminate up to 99% of the spectra representative of controls and 92% of spectra representative of disease, and for plasma, 96% of controls and 92% of disease. Several spectral wavenumbers have been identified as key biomarkers; each demonstrating significant statistical differences in band intensities when comparing subjects from the CVID and control groups. These biomarkers have been tentatively assigned to bond vibrations found in important biochemical moieties that should be further explored in relation to pathophysiological mechanisms causing CVID. This work therefore opens the way for the first application of FTIR spectroscopy in a clinical immunology laboratory, which could rapidly translate into a point-of-care device to enable ‘while-you-wait’ diagnostic testing in the immunodeficiency clinic.

## Materials and Methods

### Population

This study included 21 adult (>18 years old) CVID patients and 30 healthy age-matched controls recruited at Royal Preston Hospital. This study was approved by the ethics committee of the NHS Research Ethics Committee, Health Research Authority (HRA) (IRAS No. 212518). All samples were collected with informed written consent for study participation and all methods were carried out in accordance with relevant guidelines and regulations. Double-blinded unbiased acquisition of spectra was performed from all 51 samples following the allocation of a randomised unique study number to each subject at the point of recruitment. All patients clinically diagnosed with CVID fulfilled the European Society for Immunodeficiencies and the Pan American Group for Immunodeficiency (ESID/PAGID) (1999) diagnostic criteria^14^. The cohort characteristics are shown in Table 1 and more detailed patient demographics information is shown in Supplemental Table 1 [see Supplementary Information].

**Table 1:** Summary of CVID patient demographics.

CVID patient demographics	
**Clinical complications**	n=
Bronchiectasis	10
Splenomegaly	6
AI	4
malignancy	5
ENT	4
GI	4
Passed away	2
**Treatment**	
IVIG	6
SC	13
NONE	2
**IgG measurement (g/L)**	
IgG levels < 6	6
IgG levels > 6	15
**Age segregation (yrs)**	
20–40	6
40–60	8
60+	7
**Sex**	
Female	9
Male	12

### Immunoglobulin (IG) therapy

Management of CVID patients with immunoglobulin (IG) replacement was noted for the analysis the patient cohort results (see Supplemental Table 1). Serum immunoglobulin levels were measured for all patients and controls on the date of recruitment into the study, (IG-treated patient mean = 7.86 g/L, SD2.37; HC mean IgG = 10.48 g/L, SD = 1.72). Pre-treatment mean IgG levels for the patient cohort were 2.17 g/L, SD = 2.56.

### Clinical sub-groups

The CVID patient cohort was sub-grouped based on the presence, or absence of additional clinical complications using data extracted from individual patient files, clinical notes, and laboratory test results. The CVID-complications group consisted of 13 patients in total. Each patient in this group had documented clinical history of one or more of the following complications: bronchiectasis (*n* = 10), autoimmunity (*n* = 4), splenomegaly (*n* = 6), malignancy (*n* = 5), or gastrointestinal complications (*n* = 4). The CVID complications-free group consisted of 8 patients; review of the available clinical history (clinical letters, patient discussion, electronic/paper notes) revealed no indication of any relevant clinical complications.

### Sample Collection

Whole-blood samples were collected into EDTA-treated or serum gel-separator tubes and centrifuged at 110 × g for 5 min to separate the plasma or serum supernatant from the cells. Serum and plasma samples were then stored as 0.3 mL aliquots at −80 °C until required. Prior to spectroscopic analysis, individual aliquots were thawed; mixed and 50 µL from each aliquot was deposited onto IR-reflective glass slides (MirrIR Low-E slides; Kevley Technologies) in duplicate. Slides were left to air dry for up to 8 hours before being placed into a desiccator overnight. Once generated, dried blood spot slides were analysed the subsequent day. This process was undertaken for both serum and plasma samples.

### ATR-FTIR spectral acquisition

The spectra were obtained using a Tensor 27 FTIR spectrometer with Helios ATR attachment (Bruker Optics Ltd) operated by OPUS 5.5 software. The sampling area, defined by the internal reflection element (a diamond crystal), was ≈250 × 250 μm. Spectral resolution was 8 cm^−1^ with two times zero-filling, giving a data spacing of 4 cm^−1^ over the range 4,000–400 cm^−1^.

The acquisition of an FTIR spectrum involves collecting a ‘single-beam’ spectral measurement at one point within a sample. For each study subject, blood spots of both serum and plasma were produced in duplicate; 10 spectra were collected per 50 µL dried blood spot (total of 20 spectra per biofluid). In order to enlarge the area of acquisition and minimize bias associated with sample thickness and molecular heterogeneity, spectra were collected from 10 different point locations within each blood spot. In consideration of the well-described ‘coffee ring effect’^69–71^, point spectra from the peripheral edges of the dried blood spots were avoided. The diamond crystal was cleaned with distilled water and dried between samples and replicates. Pre-processing of spectra was performed according to recommended protocols^19,36,72^.

### Computational Analysis

The spectra files were pre-processed using the IRootLab toolbox (trevisanj.github.io/irootlab/), within MATLAB R2017a software (MathWorks). Initially, the 20 replica spectra per sample were averaged in order to work with a sample-based classification. Two pre-processing techniques were independently tested: (1) rubber-band baseline correction followed by vector normalisation^72^ and (2) by Savitzky-Golay (SG) smoothing (second-order polynomial and nine filter coefficients)^73^. Once the spectra had been pre-processed, two regions of interest were extracted from the spectra; the Fingerprint region, which covers the area between wavenumbers 1800–900 cm^−1^; and the High region, which covers wavenumbers 3700–2800 cm^−1^.

Principal component analysis linear discriminant analysis (PCA-LDA) was used to observe inter-group differences by means of a linear discriminant function applied to the principal component analysis (PCA) scores^74^. PCA is an unsupervised classification technique of exploratory analysis that reduces the spectral dataset into a small number of principal components (PCs) responsible for the majority of the original data variance^75^.

Support vector machine (SVM) is a supervised machine-learning was applied for classifying data. The data was pre-processed as above using SG-2^nd^ differential baseline correction and de-noising and vector normalisation. We used an SVM algorithm performed in MATLAB, run with an n-fold leave one out, cross validation technique (n = 5) to select the best parameters for *c* and gamma (*γ)*. The parameters (c, γ) for SVM are selected by using a grid search function in MATLAB^72^. To investigate the classification rate, sensitivities and specificities were calculated for each model tested^76^. The SVM was trained using 2/3 of the spectral data and tested using the remaining 1/3. The data set was split using the Kennard-Stone algorithm to achieve uniformity and representativeness within the samples selected for the training set^77^. This splitting process was performed in a patient basis, where the spectral data assigned to the training and test sets were from different samples, so the training and test groups do not contain spectra from the same patient. The models were built using 10-fold cross-validation for optimization. The classification percentage calculated from the confusion balls (graphical representation of a confusion matrix) of each SVM model designates the rate of correct group assignation when applying the test dataset to the trained SVM model. Sensitivity and specificity of each SVM classification was calculated using the accumulative hits data (number of true positives, true negatives, false positives, and false negatives) generated from the confusion matrices.

Feature extraction was performed on the training dataset to extract potential biomarkers and identify the spectral wavenumbers that account for the largest differences between the CVID and HC groups. This was undertaken using three methods of biomarker extraction on the training dataset for serum and plasma: Student’s T-Test, PCA-LDA and Feature Forward Selection (FFS), for both Fingerprint and High regions of the spectra. The six key biomarkers extracted from each method were subsequently investigated for relative increases or decreases in absorbance intensity between the classes (subject groups). Wavenumbers not demonstrating significant intensity variance between CVID and HC groups were not taken forward for individual subject level intensity analysis (using average intensities of 20 spectral replicates). Extracted wavenumbers within close proximity (10 cm^−1^) of an adjacent biomarker were omitted, as closely associated wavenumbers will be influenced from intensity increases or decreases in nearby peaks already identified as biomarkers.

The Student’s T-Test method was performed on the training dataset for both fingerprint and high regions of the spectra. The −log10 of the P-value of the T-test for each wavenumber was then plotted to identify the potential biomarkers from the T-test. The biomarkers extracted following PCA-LDA were obtained from the cluster vector analysis. FFS was applied within IRootLab using the PCA loadings to identify the main biomarkers responsible for class segregation by calculating p-values for the variables with larger loadings coefficients^78^. A peak detection algorithm was applied to each method to identify the six most segregating peaks. Extracted wavenumbers within 10 cm^−1^ proximity of an adjacent biomarker were also omitted (n = 2) resulting in a total of 10 spectral wavenumbers (p =< 0.05).

### Statistical analysis

A student’s t-test (two–tailed, 95% confidence interval (CI)) was performed to calculate statistical significance of spectral variance between groups, with a P-value of less than 0.05 being considered significant. A power test based on a two-tailed t-test (data input as mean and standard-deviation of the plasma pre-processed spectra in the fingerprint region for each class) indicated a minimum number of samples of 26 HC and 15 CVID patients for a power of 80%. The number of samples used herein (HC = 30, CVID = 21) is above this minimum. Statistical analysis was carried out on averaged spectra to account for differences between individuals and not spectra.

### Data deposition

The data (raw spectra and pre-processed spectra) reported in this paper are available at the publicly accessible data repository Figshare (10.6084/m9.figshare.7751309).

## Supplementary information

Supplementary information

## Data Availability

All data (raw and pre-processed spectra) along with appropriate code identifiers will be uploaded onto the publicly accessible data repository Figshare.
