# Supplementary material for: New approach to investigate Common Variable Immunodeficiency patients using spectrochemical analysis of blood
Source: Sci Rep. 2019 May 10;9:7239. doi: 10.1038/s41598-019-43196-5 (PMC6510896; doi:10.1038/s41598-019-43196-5)
Supplement: Supplementary file 1 — Supplementary information [file 41598_2019_43196_MOESM1_ESM.docx]

**New approach to investigate Common Variable Immunodeficiency patients using spectrochemical analysis of blood**

**Emma L. Callery,*^a^ Camilo L. M**. **Morais,^b^ Maria Paraskevaidi,^b e^ Vladimir Brusic,^c^ Pavaladurai Vijayadurai,^a^ Ariharan Anantharachagan,^a^ Francis L. Martin, ^b^ & Anthony W. Rowbottom^*a d^**

**Affiliations**

*Corresponding authors

^a^ Department of Immunology, Lancashire Teaching Hospitals NHS Foundation, Preston PR2 9HT, UK

^b^ School of Pharmacy and Biomedical Sciences, University of Central Lancashire, Preston PR1 2HE, UK

^c^ University of Nottingham Ningbo China, Ningbo 315100, China

^d^ School of Medicine, University of Central Lancashire, Preston PR1 2HE, UK

^e^ Department of Surgery and Cancer, Imperial College London, W12 0HS

**Supplementary Information**

**Supplemental Figure 1 – Improving robustness, accuracy and interpretability of the data. Class means of pre-processing techniques applied to all spectra in training dataset (CVID *n*=13 (260 spectra); HC *n*=18 (360 spectra)) to correct for experimental variation and to improve resolution of peak overlap. For each subject 20 individual spectra were acquired per biofluid (10 replicates of each dried blood spot, in duplicate). a-d Fingerprint region (900-1800 cm^-1^). a, b, Rubber-band corrected, vector normalised spectra for serum and plasma respectively. c, d, Second order differentiated, vector normalised spectra for serum and plasma respectively. e-h, High region (2800-3700 cm^-1^). Rubber-band corrected, vector normalised spectra for serum and plasma respectively. g, h, Second order differentiated, vector normalised spectra for serum and plasma respectively.**

| **Serum Fingerprint region** | **Plasma Fingerprint region** |
| --- | --- |
|  |  |
| 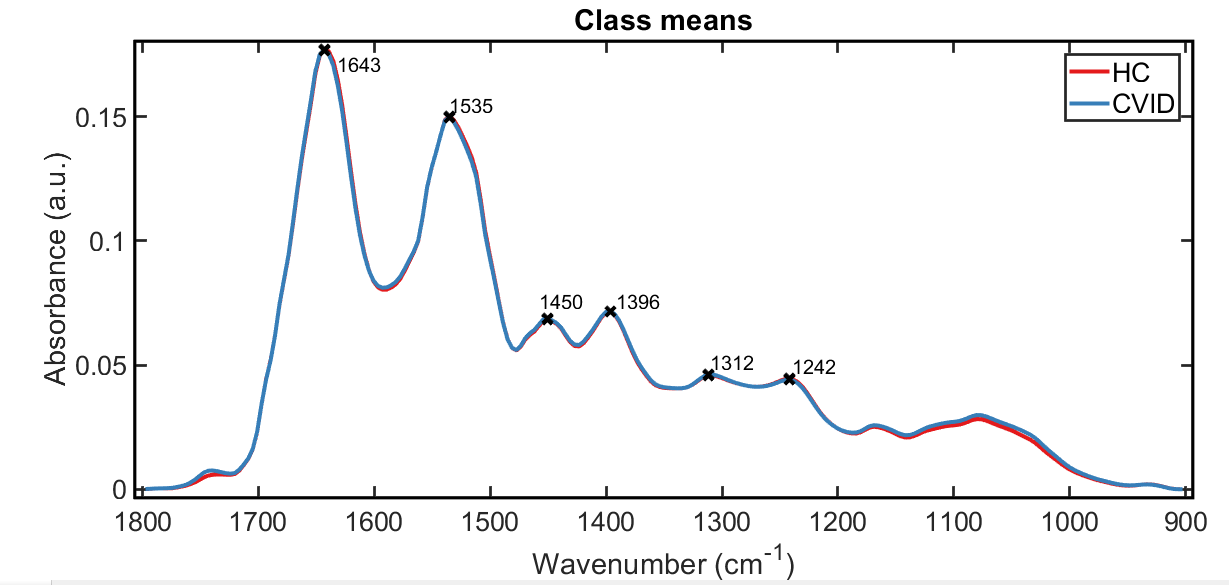 | 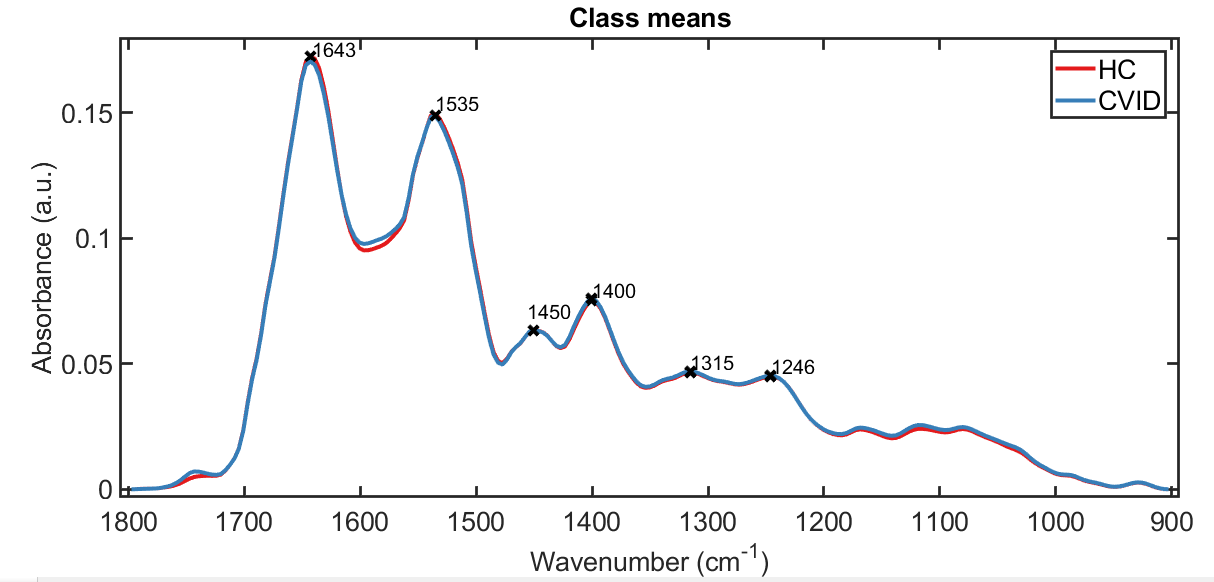 |
|  |  |
| 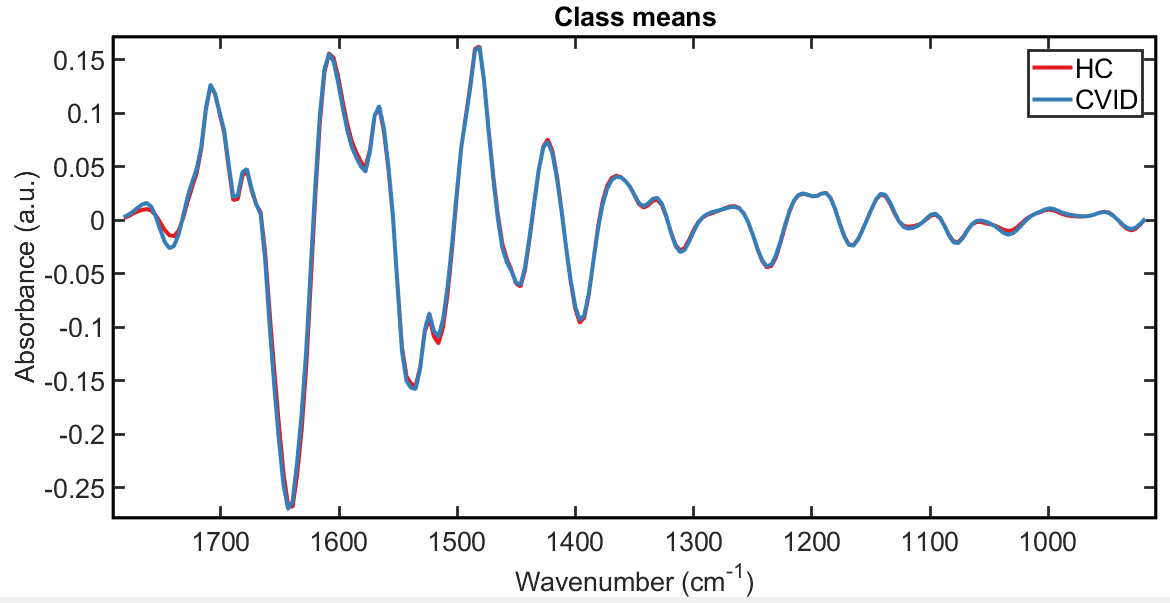 | 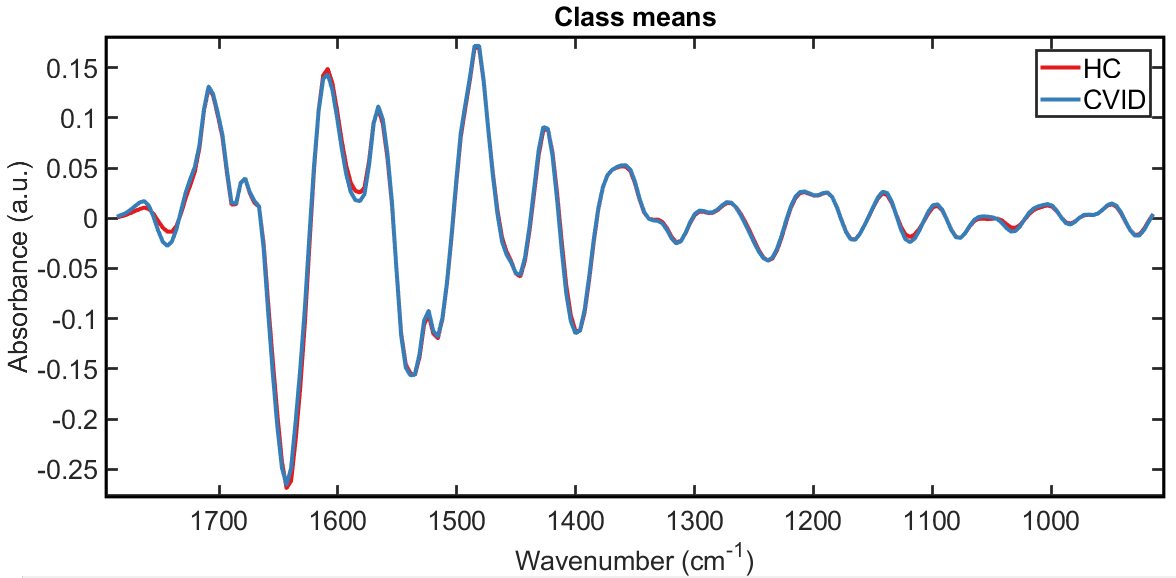 |
| **Serum High Region** | **Plasma High Region** |
|  |  |
| 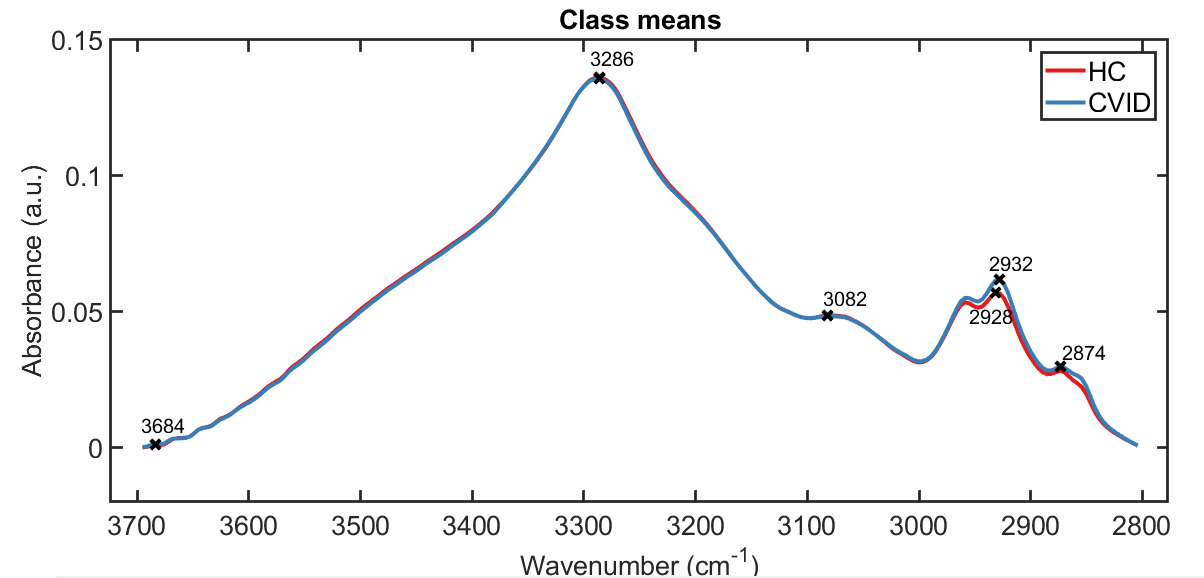 | 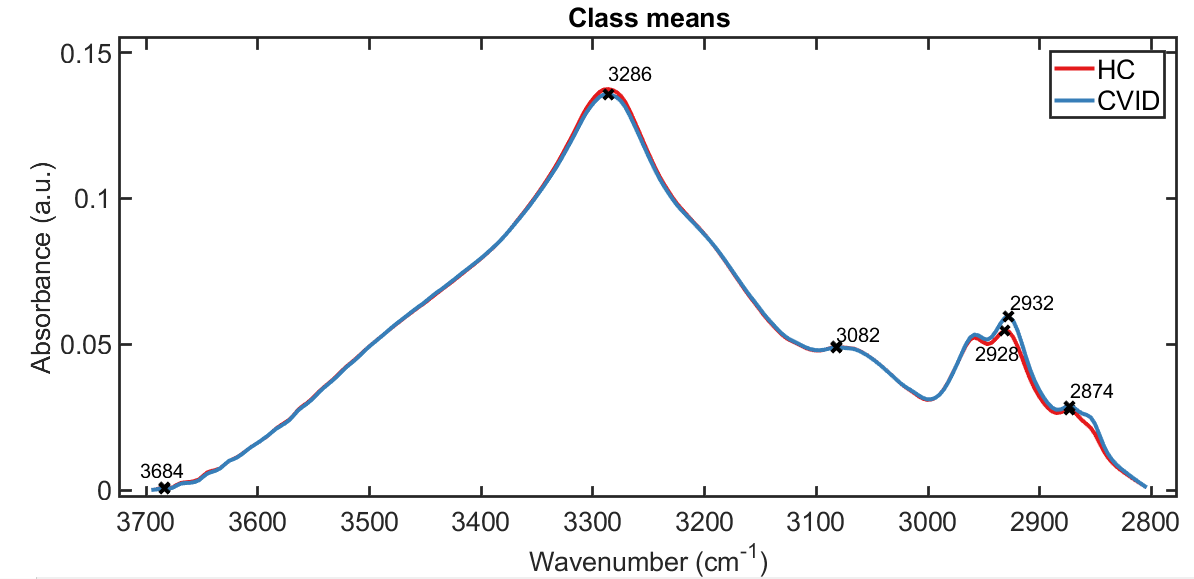 |
|  |  |
| 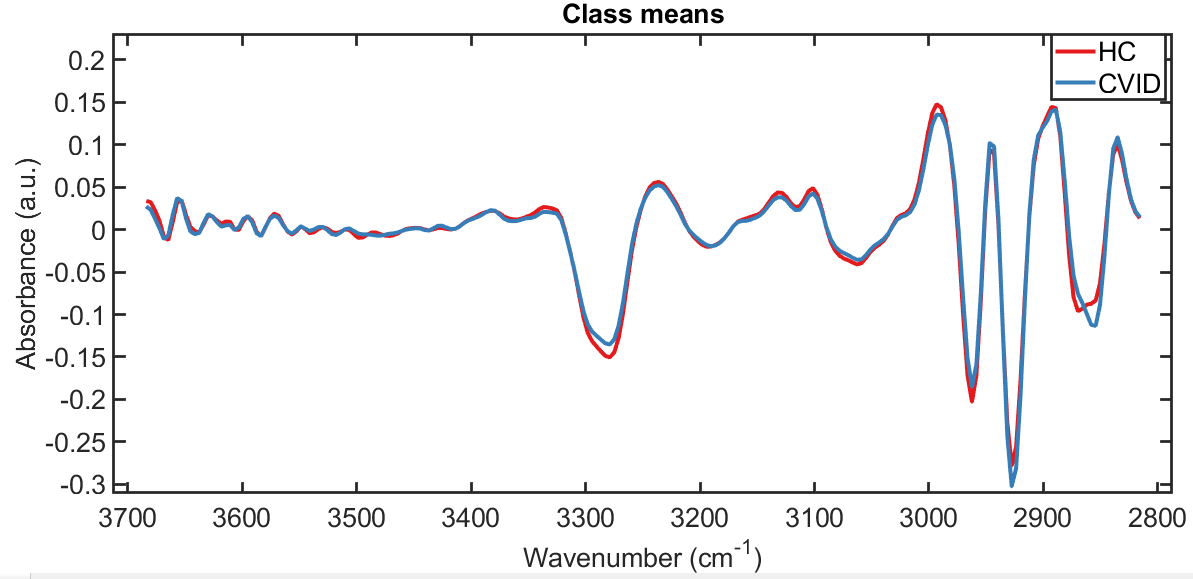 | 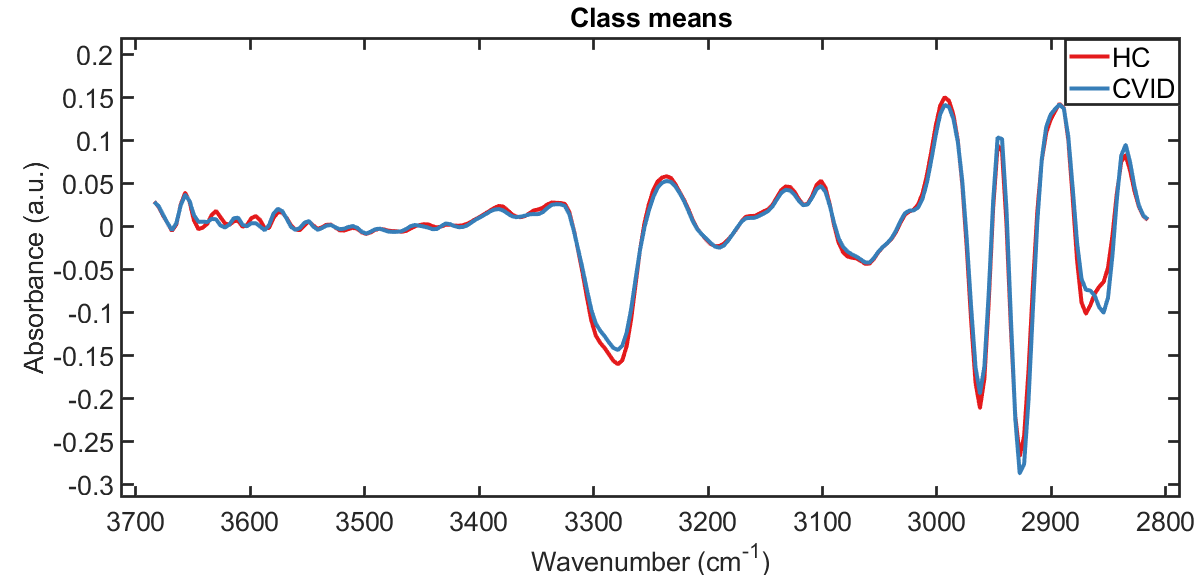 |

**Supplemental Figure 2 - Supervised multivariate analysis techniques (PCA-LDA) successfully segregate classes on a subject level basis (CVID vs HC). a, b, Fingerprint region (900-1800 cm^-1^); 1D scores plots (LD1) after cross-validated PCA-LDA of the training dataset (CVID *n*=13; HC *n*=18) for serum and plasma respectively. c,d, High region (2800-3700 cm^-1^); 1D scores plots (LD1) after cross-validated PCA-LDA of the training dataset (CVID *n*=13; HC *n*=18) for serum and plasma respectively.**

| **Serum Fingerprint Region** | **Plasma Fingerprint Region** |
| --- | --- |
| 1. Serum fingerprint region cross-validated PCA-LDA scores plots (p <0.0005) | 1. Plasma fingerprint region cross-validated PCA-LDA scores plots (p <0.0005) |
| **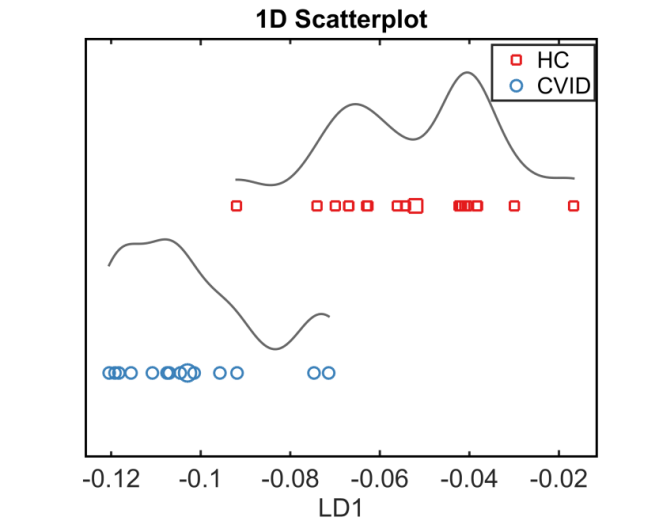** | **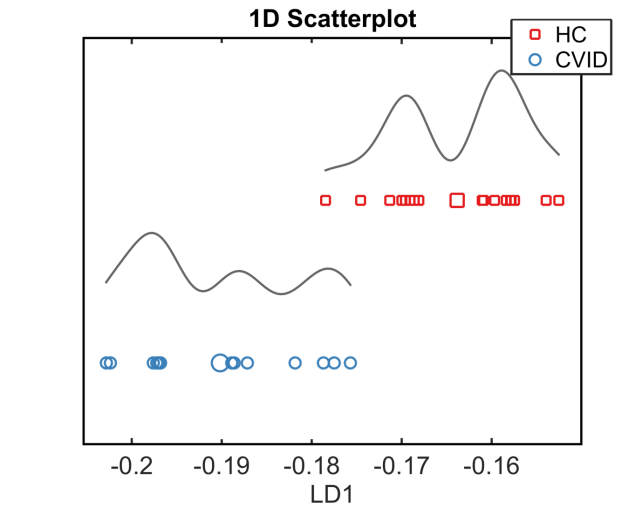** |
| **Serum High Region** | **Plasma High Region** |
| 1. Serum high region cross-validated PCA-LDA scores plots (p <0.0005) | 1. Plasma high region cross-validated PCA-LDA scores plots (p <0.0005) |
| **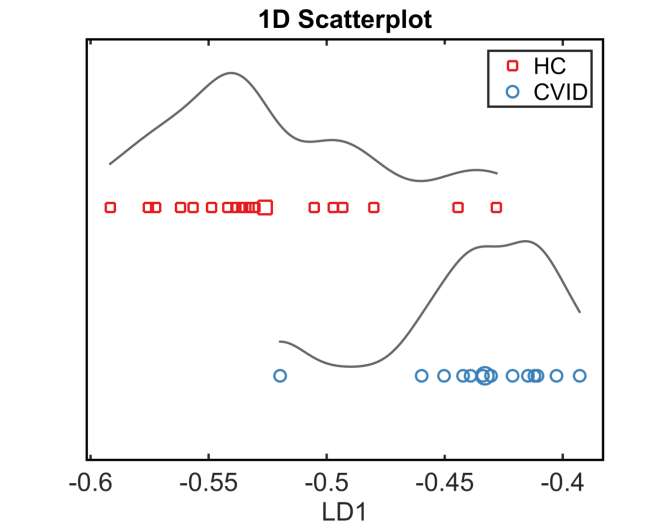** | **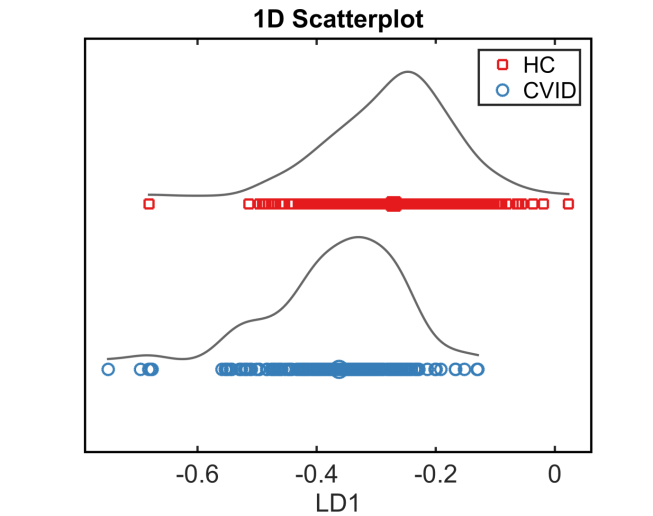** |

**Supplemental Figure 3. - Serum and plasma High region biomarkers identified using three feature extraction methods. a, Serum T-test. b, Plasma T-test. c, Serum FFS. d, Plasma FFS. e, Serum cross-validated PCA-LDA. f, Plasma cross-validated PCA-LDA. i, j, Visual representation of wavenumber location for extracted biomarkers from each method for serum and plasma respectively. FFS Forward Feature Selection.C HC HVCH**

| **Serum High Region** | **Plasma High Region** |
| --- | --- |
| 1. T-test | 1. T-test |
| 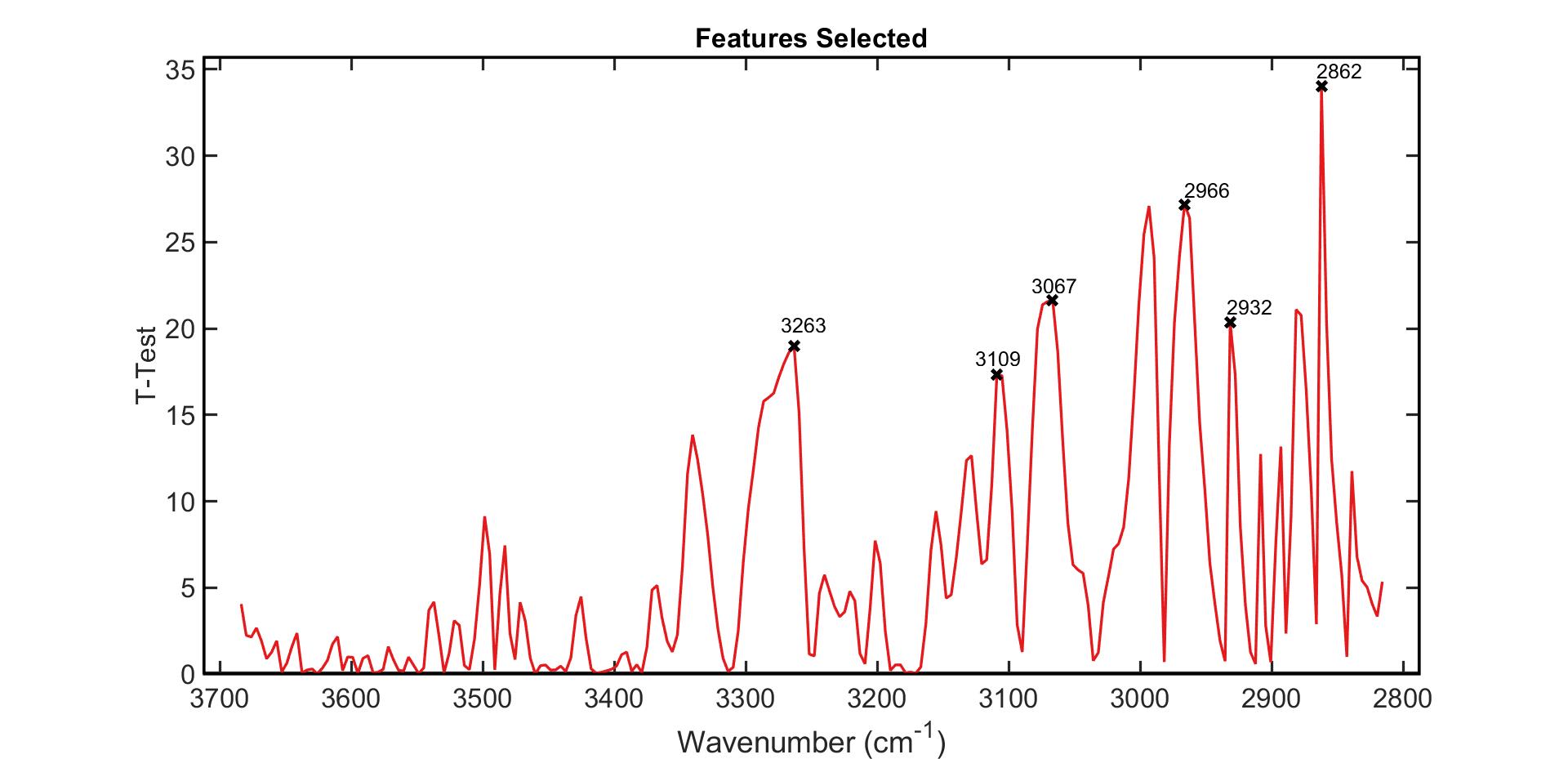 | 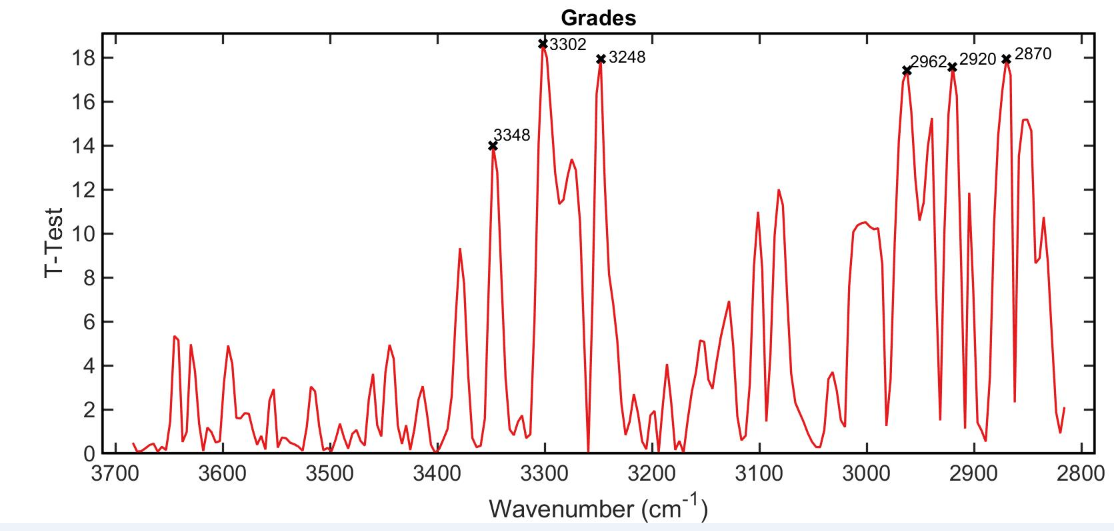 |
| 1. PCA-LDA Cluster Vectors | 1. PCA-LDA Cluster Vectors |
| 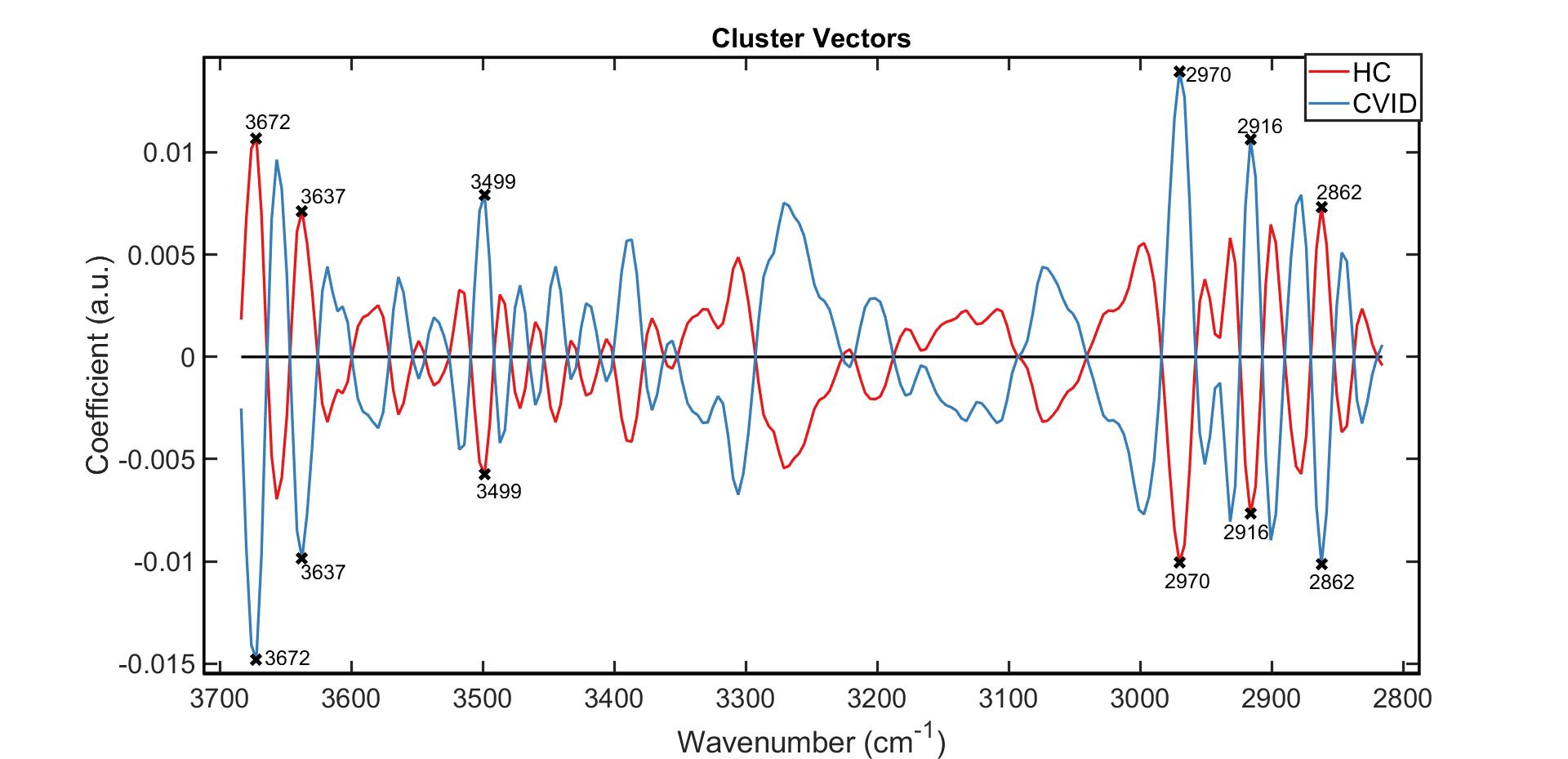 | 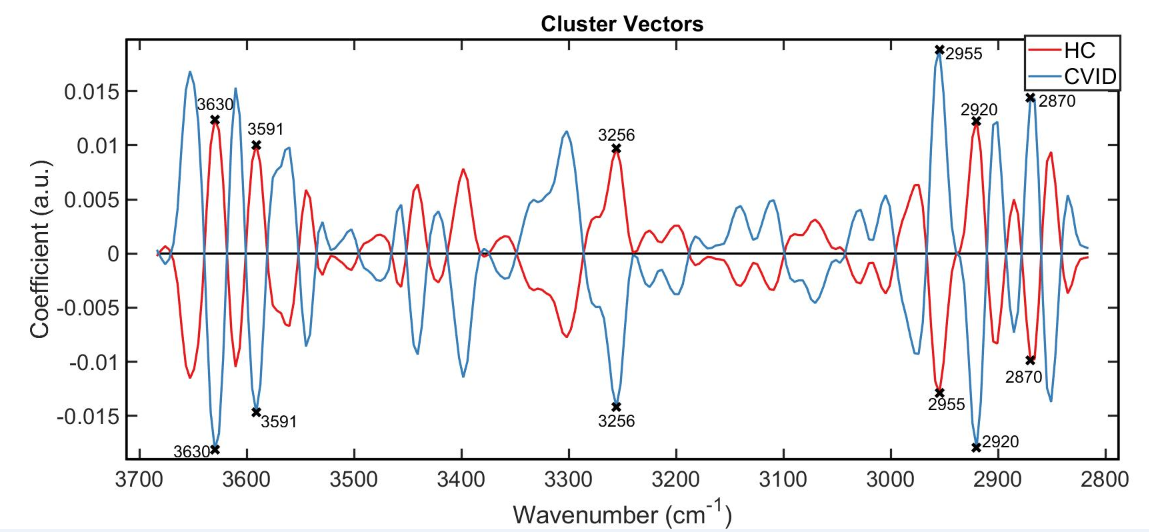 |
| 1. FFS | 1. FFS |
| 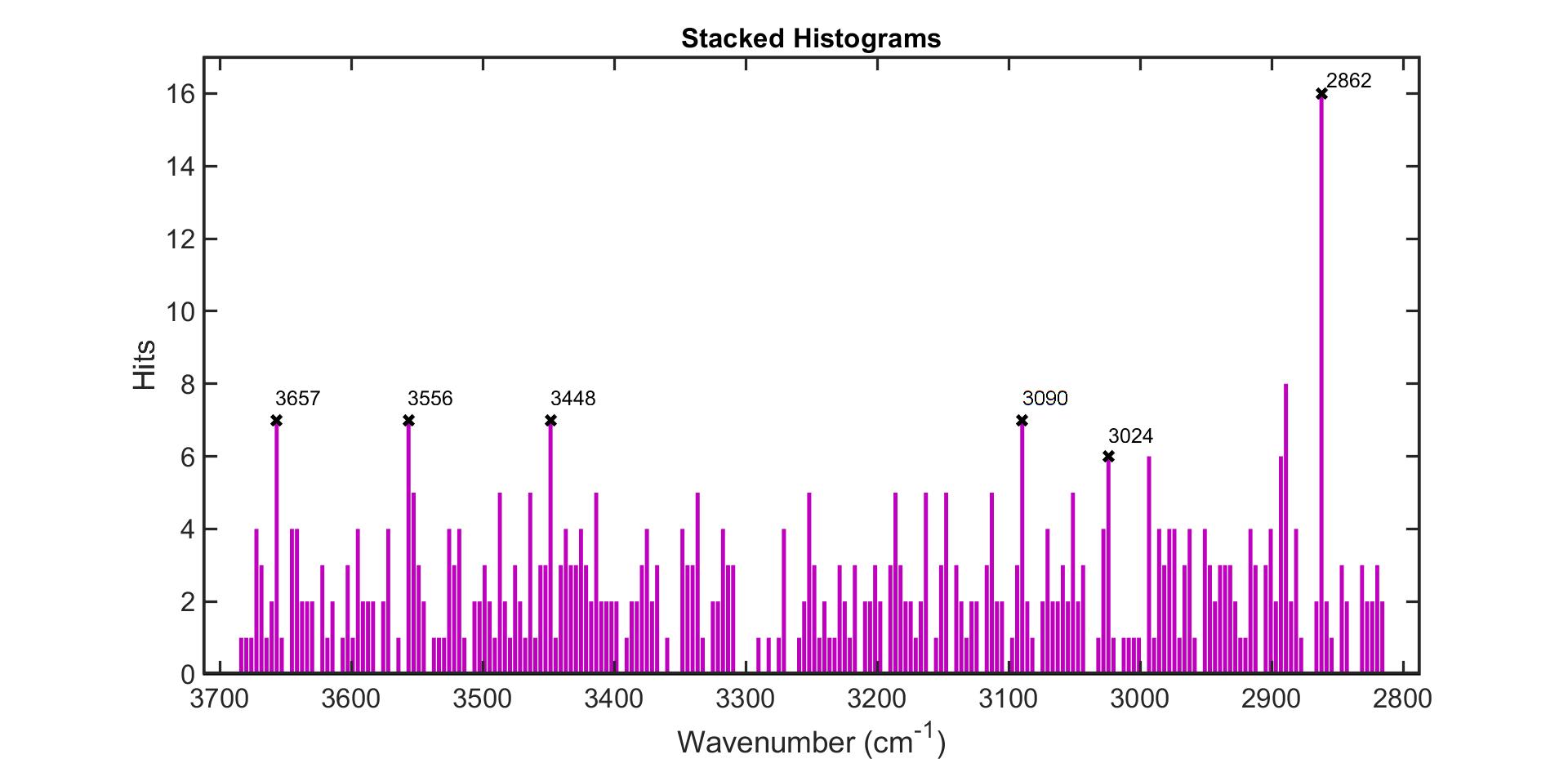 | 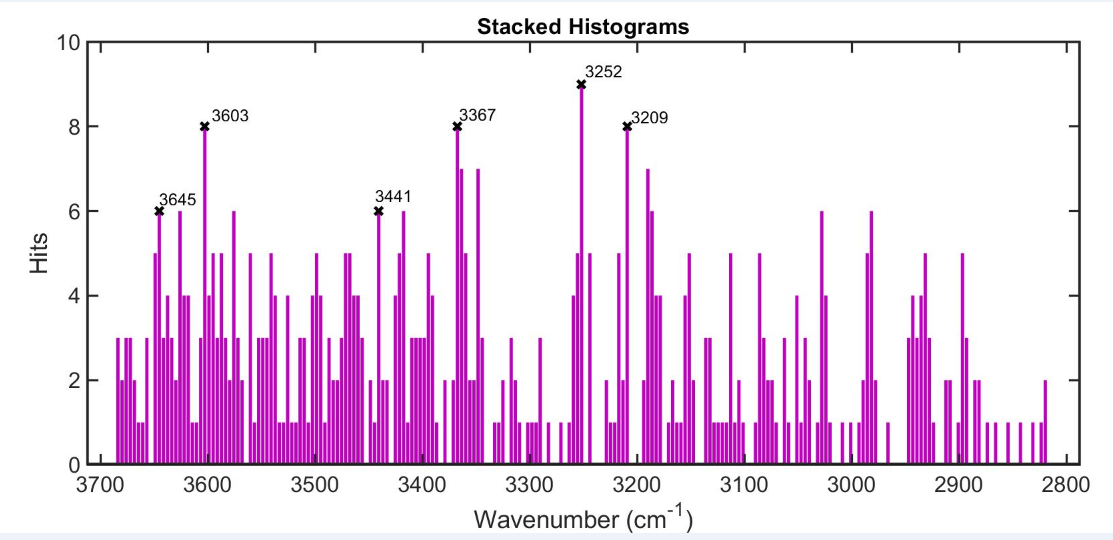 |
| 1. Comparison of all extracted wavenumbers | 1. Comparison of all extracted wavenumbers |
| 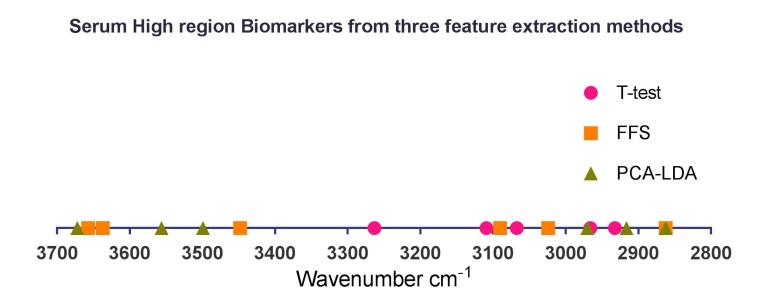 | 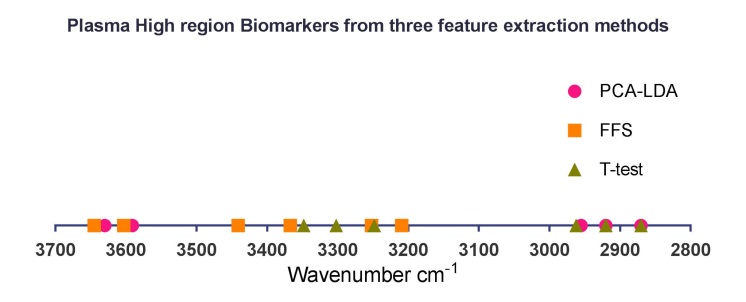 |

**Supplemental Table 1. Patient demographics**

| Study No. | Sex | Age (y) | B cells | MemB | CSM | IV/ SC | Dose (g) | last infused | Days post infusion | monthly/weekly | Pre-tx IgG | Dx (years) | Immunoglobulin level at recruitment | | | Spenomegaly | Autoimmunity | Bronciectasis | Malignancy | ENT | GI | Stable/ Improved/Deteriorating |
| --- | --- | --- | --- | --- | --- | --- | --- | --- | --- | --- | --- | --- | --- | --- | --- | --- | --- | --- | --- | --- | --- | --- |
|  |  |  |  |  |  |  |  |  |  |  |  |  | IgG | IgA | IgM |  |  |  |  |  |  |  |
| 65639 | M | 22 | 576 | 28 | 8 | SC | 5.25 | 26/02/2017 | 2 | weekly | 2.6 | 3 | 9.8 | 0 | 0.3 | No | No | No | No | No | No | Stable |
| 39591 | M | 21 | - Not done - | | | IV | 30 | 03/01/2017 | 21 | 3-weekly | 0.52 | 8 | 6.7 | 0 | 0.01 | Yes | Diabetes | No | No | No | No | Stable (passed away) |
| 75983 | F | 62 | 267 | 2 | 0 | IV | 25 | 03/01/2017 | 28 | 3-weekly | 0.38 | 13 | 10.4 | 0 | 0.03 | No | No | No | No | Yes | No | Stable |
| 53349 | M | 70 | 200 | 10 | 0 | SC | 8 | 18/01/2017 | 6 | weekly | 0.77 | 5 | 7.3 | 0 | 0.38 | Splenectomy | No | Yes | No | Infecti-ons | Diarrhoea | Stable |
| 81275 | F | 51 | 295 | 15 | 2 | N/A | - | - | - | - | 4.53 | 3 | 3.8 | 0.17 | 2.22 | Splenectomy 1988 | Haemolytic anaemia 1999 | No | Bladder | No | No | Stable |
| 70913 | F | 26 | 423 | 3 | 0 | SC | 6 | 19/02/2017 | 2 | weekly | 0.2 | 6 | 5.3 | 0 | 0.02 | No | No | No | No | No | No | Stable |
| 90802 | M | 78 | 24 | 42 | 33 | SC | 12 | 02/05/2017 | 6 | weekly | 2.35 | 12 | 7.5 | 0.87 | 0.18 | No | No | No | No | No | No | Stable |
| 87088 | F | 53 | 27 | 5 | 0 | SC | 6 | 20/09/2017 | 6 | weekly | 0.34 | 16 | 5.9 | 0 | 0.01 | Yes | Thrombocyt-openia & neutropenia | Yes | Squamous cell (skin) | No | Yes | Deteriorated |
| 10415 | F | 56 | 240 | 15 | 1 | IV | 25 | 19/04/2017 | 6 | 3-weekly | unknown | >10 | 11.4 | 0 | 0.03 | No | No | Yes | No | Yes | No | Stable |
| 19759 | F | 20 | 50 | 16 | 7 | N/A | - | - | - | - | 1.33 | 2 | 1.3 | 0 | 0.02 | No | No | No | No | Yes | Diarrhoea | Improved |
| 98037 | M | 50 | 287 | 23 | 6 | IV | 35 | unknown | unknown | 4-weekly | 0.53 | 11 | 7.2 | 0 | 0.02 | No | No | No | No | No | No | Stable |
| 13582 | F | 79 | 64 | 18 | 10 | IV | 25 | 01/02/2017 | 28 | 4-weekly | 3.25 | 7 | 7.1 | 2.07 | 0.28 | No | No | Yes | Basal Cell | No | No | Deteriorating (Passed away) |
| 48004 | M | 33 | 28 | 83 | 79 | SC | 10 | 23/05/2017 | 0 | weekly | 1.47 | 3 | 7.8 | 0 | 0.06 | No | No | No | No | No | No | Stable |
| 73741 | M | 56 | 28 | 83 | 79 | SC | 9.6 | 21/05/2017 | 2 | weekly | 10.65 | Pre 2005 | 13.8 | 0.09 | 0.01 | No | No | No | No (MGUS) | No | No | Stable |
| 97511 | M | 80 | 39 | 15 | 6 | SC | 8 | 22/02/2017 | 6 | weekly | 3.0 | 3 | 6.47 | 0.67 | 0.33 | No | No | Yes | No | Sinus infecti-ons | No | Deteriorating |
| 50735 | F | 53 | 11 | 4 | 0 | SC | 9 | 23/07/2017 | 2 | weekly | 0.3 | 3 | 5.07 | 0 | 0 | No | No | Yes | No | No | No | Stable |
| 90326 | M | 53 | 69 | 33 | 17 | IV | 40 | 04/06/2017 | 9 | 3-weekly | unknown | >20 | 11.16 | 0 | 0 | No | No | Yes | No | No | Yes | Deteriorating |
| 29891 | M | 31 | 23 | 0 | 0 | SC | 9.9 | unknown | unknown | weekly | 0.3 | 3 | 7.89 | 0 | 0.01 | Yes | No | No | No | No | No | Stable |
| 36952 | M | 48 | 28 | 22 | 0 | SC | 6.5 | 16/01/2017 | 1 | weekly | 1.9 | 10 | 5.61 | 0 | 0.05 | Yes | Cytopenia | Yes | Orophar-yngeal | No | No | Stable |
| 31108 | M | 61 | 173 | 5 | 0 | SC | 10.4 | 24/03/2017 | 4 | 5-6 daily |  | 1 | 6.24 | 0.04 | 0.03 | No | Primary biliary cirrhosis | Yes | No | No | No | Improved |
| 61236 | F | 72 | 125 | 4 | 2 | SC | 6 | unknown | unknown | weekly | 4.7 | 12 | 6.71 | 0.44 | 0.05 | No | No | Yes | Breast (2008) | No | No | Stable |

**Supplemental Table 2. Parameters for the SVM models. Classification of HC vs CVID.**

|  | **Serum** | | **Plasma** | |
| --- | --- | --- | --- | --- |
| Grid-search parameters | *c* | γ | *c* | γ |
| Fingerprint region | 1 x 10^7 | 1x 10^-1 | 1 x 10^7 | 1x 10^-1 |
| High region | 1 x 10^3 | 1x 10^1 | 1 x 10^3 | 1x 10^1 |

**Supplemental Table 3. Parameters for the SVM models following sub-classification of CVID patients. HC vs CVID-non complications vs CVID-complications.**

|  | **Serum** | | **Plasma** | |
| --- | --- | --- | --- | --- |
| Grid-search parameters | *c* | γ | *c* | γ |
| Fingerprint region | 1 x 10^-1 | 1x 10^-7 | 1 x 10^3 | 1x 10^-1 |
| High region | 1 x 10^7 | 1 x 10^-2 | 1 x 10^5 | 1 x 10^-1 |

**Supplemental Table 4. Serum & plasma fingerprint wavenumbers.**

| Wavenumber (cm^-1^) | Biofluid | Tentative Assignments | Reference | Method | P-value (T-Test) | ↑↓ In CVID |
| --- | --- | --- | --- | --- | --- | --- |
| 933 | Serum | Z type DNA | ^64^ | T-Test | 0.0203* |  |
| 984 | Plasma | Phosphodiester region (900-1300 cm^-1^) | ^51,65^ | FFS | 0.000469 | **↑** |
| 1007 | Plasma | Ring stretching vibrations mixed strongly with CH in-plane bending (1000-50 cm^-1^) | ^66^ | T-Test | 3.15E-25 | **↑** |
| 1034 | Serum | Collagen | ^42^ | T-Test & PCA-LDA | 1.31E-12**** | **↑** |
| 1053 | Plasma | vC - O & dC - O of carbohydrates, Shoulder of 1121 cm^-1^ (Symmetric phosphodiester stretching band)  DNA (nucleic acids and phospholipids) | ^42,57,58^ | FFS, T-test | 2.64E-69 | **↑** |
| 1084 | Plasma | DNA (band due to PO_2_^-^ vibrations), Nucleic acid–Phosphate band | ^44,45,54–58^ | PCA-LDA | 0.007575 | **↑** |
| 1107 | Plasma | v(CO), v(CC), ring (polysaccharides, pectin) | ^83^ | FFS | 2.74E-05 | **↑** |
| 1107 | Serum | v(CO), v(CC), ring (polysaccharides, pectin) | ^83^ | FFS | 2.53E-20**** |  |
| 1115 | Serum | Symmetric stretching P – O – C | ^43^ | T-Test | 2.02E-26**** | **↑** |
| 1119 | Plasma | C - O stretching mode | ^84^ | T-Test | 6.94E-30 |  |
| 1312 | Serum | Amide III band components of proteins | ^85,86^ | T-Test | 1.49E-08**** |  |
| 1393 | Serum | CH_2_ wagging vibration of the acyl chains (phospholipids) (1250-400 cm^-1^) | ^42^ | FFS | 4.23E-06**** |  |
| 1416 | Plasma | Deformation C–H, N–H, stretching C–N | ^43^ | PCA-LDA | 8.34E-13 | **↑** |
| 1420 | Serum | Ring stretching vibrations with CH in-plane bending (1400-500 cm^-1^) | ^87^ | PCA-LDA | 8.73E-07**** |  |
| 1435 | Plasma | Ring stretching vibrations with CH in-plane bending (1400-500 cm^-1^) | ^87^ | T-Test | 1.43E-28 |  |
| 1528 | Serum | C=N guanine, adenine, cytosine | ^43,44^ | PCA-LDA | 5.57E-12**** | **↓** |
| 1566 | Plasma | Amide II (1540-650 cm^-1^) | ^88^ | FFS | 0.004467 | **↑** |
| 1589 | Serum | Amide II (1540-650 cm^-1^) | ^88^ | FFS | 0.00109** |  |
| 1639 | Plasma | C=C thymine, adenine, N-H guanine  Amide I | ^43,85^ | T-Test | 3.08E-39 | **↓** |
| 1651 | Serum | Amide I: (mainly protein C=O stretching), α-helical structure | ^80,89^ | T-Test | 0.0462* |  |
| 1732 | Plasma | C=O stretching band mode of the fatty acid ester (1725-45 cm^-1^) | ^90^ | T-Test | 1.09E-19 |  |
| 1759 | Serum | C=O vibrations of esters (triglycerides) | ^45,91^ | FFS | 7.61E-12**** | **↑** |
| 1763 | Plasma | Fatty acid esters (1700-1800 cm^-1^) | ^90^ | FFS | 1.30E-13 |  |

*Significant to *P <0.05; **P <0.005; ***P 0.0005; ****P <0.00005.*

**Supplemental Table 5. Serum and Plasma High region biomarkers.**

| Wavenumber (cm^-1^) | Biofluid | Tentative Assignments | Reference | Method | P-value |
| --- | --- | --- | --- | --- | --- |
| 2862 | Serum | Fatty acids | ^89^ | T-Test/PCA-LDA/FFS | 1.19E-13**** |
| 2870 | Plasma | CH_3_ symmetric stretching: protein side chains, lipids, with some  contribution from carbohydrates and nucleic acids | ^80^ | T-Test, PCA-LDA | 1.13E-18 |
| 2916 | Serum | vibrations of CH_2_ and CH_3_ of phospholipids, cholesterol and creatinine | ^34^ | PCA-LDA | 2.79E-10**** |
| 2920 | Plasma | C–H, Lipid region,  CH_3_, CH_2_-lipid and protein (2800-3000 cm^-1^) | ^42,92,93^ | T-Test, PCA-LDA | 2.64E-18 |
| 2932 | Serum | C-H, C-H stretching bands in malignant tissues | ^46,92^ | T-Test | 3.99E-12**** |
| 2962 | Plasma | CH_3_ asymmetric stretching | ^94^ | T-Test | 3.72E-18 |
| 2970 | Serum | ν_as_ CH_3_, lipids, fatty acids | ^83^ | PCA-LDA | 1.53E-05**** |
| 3024 | Serum | C-H stretching vibrations of methyl (CH_3_) and methylene (CH_2_)  groups and olefins (2800–3100 cm^-1^) | ^46^ | FFS | 0.0003*** |
| 3067 | Serum | C-H stretching vibrations of methyl (CH_3_) and methylene (CH_2_)  groups and olefins (2800–3100 cm^-1^) | ^46^ | T-Test | 0.00714* |
| 3248 | Plasma | Symmetric and asymmetric vibrations attributed to water. So, it would be better not to consider this region for detailed  analysis (3200-550 cm^-1^) | ^34^ | T-Test | 1.14E-18 |
| 3302 | Plasma | As above | ^34^ | T-Test | 2.31E-19 |
| 3348 | Plasma | As above | ^34^ | T-Test | 0.000206 |
| 3441 | Plasma | As above | ^34^ | FFS | 5.05E-05 |
| 3448 | Serum | As above | ^34^ | FFS | 2.72E-08**** |
| 3499 | Serum | As above | ^34^ | PCA-LDA | 0.0146* |
| 3556 | Serum | OH bonds(3500–600 cm^-1^) | ^95^ | PCA-LDA | 0.00108** |
| 3591 | Plasma | OH bonds(3500–600 cm^-1^) | ^95^ | PCA-LDA | 8.48E-05 |
| 3630 | Plasma | O-H stretching (water) | ^42^ | PCA-LDA | 1.10E-05 |
| 3637 | Serum | O-H stretching (water) (3000–700 cm^-1^) | ^42^ | FFS | 0.00019** |
| 3645 | Plasma | O-H stretching (water) (3000–700 cm^-1^) | ^42^ | FFS | 4.60E-06 |
| 3672 | Serum | O-H stretching (water) (3000–700 cm^-1^ ) | ^42^ | PCA-LDA | 0.032* |

*Significant to *P <0.05; **P <0.005; ***P <0.0005; ****P <0.00005.*
